# Frank-Starling mechanism, fluid responsiveness, and length-dependent activation: Unravelling the multiscale behaviors with an in silico analysis

**DOI:** 10.1371/journal.pcbi.1009469

**Published:** 2021-10-11

**Authors:** Sarah Kosta, Pierre C. Dauby

**Affiliations:** GIGA–In Silico Medicine, University of Liège, Liège, Belgium; Stanford University, UNITED STATES

## Abstract

The Frank-Starling mechanism is a fundamental regulatory property which underlies the cardiac output adaptation to venous filling. Length-dependent activation is generally assumed to be the cellular origin of this mechanism. At the heart scale, it is commonly admitted that an increase in preload (ventricular filling) leads to an increased cellular force and an increased volume of ejected blood. This explanation also forms the basis for vascular filling therapy. It is actually difficult to unravel the exact nature of the relationship between length-dependent activation and the Frank-Starling mechanism, as three different scales (cellular, ventricular and cardiovascular) are involved. Mathematical models are powerful tools to overcome these limitations. In this study, we use a multiscale model of the cardiovascular system to untangle the three concepts (length-dependent activation, Frank-Starling, and vascular filling). We first show that length-dependent activation is required to observe both the Frank-Starling mechanism and a positive response to high vascular fillings. Our results reveal a dynamical length dependent activation-driven response to changes in preload, which involves interactions between the cellular, ventricular and cardiovascular levels and thus highlights fundamentally multiscale behaviors. We show however that the cellular force increase is not enough to explain the cardiac response to rapid changes in preload. We also show that the absence of fluid responsiveness is not related to a saturating Frank-Starling effect. As it is challenging to study those multiscale phenomena experimentally, this computational approach contributes to a more comprehensive knowledge of the sophisticated length-dependent properties of cardiac muscle.

## Author summary

It is commonly admitted that the length-dependent activation is the cellular property underlying the Frank-Starling mechanism. However, it is challenging to assess the cardiac cell length *in vivo*, and to link it to system-level variables. In this study, we investigated the relationship between length-dependent activation (LDA), the Frank-Starling mechanism, and vascular filling therapy using a multiscale model of the cardiovascular system. We showed that the Frank-Starling mechanism is a multiscale, dynamical and LDA-driven phenomenon involving essential connections between the three cellular, ventricular and cardiovascular scales. We also demonstrated that the response to vascular filling therapy is similarly multiscale and LDA-driven, and that afterload is an important factor to account for when trying to predict the system fluid responsiveness.

## 1. Introduction

**Fig 1:**
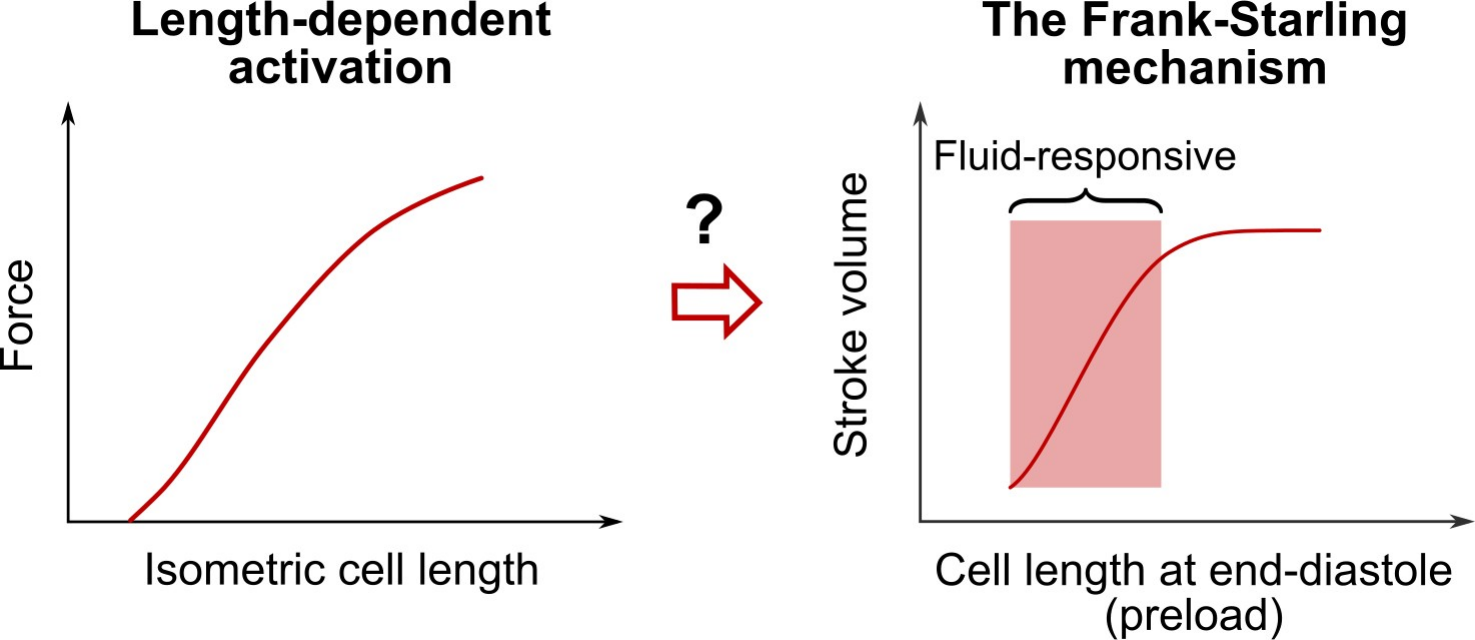
Length-dependent activation, the Frank-Starling mechanism and vascular filling therapy. Length-dependent activation (LDA) is a cardiac cell property highlighted by the length-tension relationship (left). As the length of the cardiac cell increases, the peak force during an isometric twitch contraction increases. It is commonly admitted that LDA underlies the Frank-Starling (FS) mechanism, a property of the cardiac pump (right). As the length of cardiac fibers prior to contraction (namely, the preload) increases, the ejected blood volume (namely, the stroke volume) increases, up to physiological limits. The FS mechanism is also often assumed to be the basis for fluid therapy. If the patient is operating on the “ascending portion” of the FS curve, the patient is said to be “fluid-responsive”, as a substantial stroke volume increase should be observed upon vascular filling. In this study, we revisit the three concepts of LDA, FS mechanism, and vascular filling and we build a computational model of the human cardiovascular system to investigate thoroughly the true nature of the relationship between the latter.

The Frank-Starling (FS) mechanism [1,2] is an important cardiac property addressed in every cardiology book. It states that the heart is able to adapt the ejected blood volume to the venous return on a beat-to-beat basis (see Fig 1). As more blood enters the ventricle, preload, defined as the length of cardiac fibers prior to contraction, increases. As a consequence, stroke volume increases (up to physiological limits).

To further develop Frank and Starling’s findings, many experimental studies were conducted to clarify the relationship between preload and cardiac output. For instance, Sarnoff and Berglund [3,4] explored the validity of the “Law of the Heart” in open-chest anesthetized dogs with an intact systemic circulation. They came up with the idea of a "family of Starling curves". For a fixed inotropic state, their studies indicate that there is always a direct relationship to be found between preload and cardiac output. Braunwald and colleagues studied the FS mechanism in human patients [5–9]. Working with a variety of protocols and measurements methods, they concluded that the FS mechanism is present in man. Actually, many studies confirmed this intrinsic ability of the heart to accommodate changes in preload [10], thus corroborating the early findings of Starling: "Within wide limits, the heart is able to increase its output in direct proportion to the inflow." [11].

It is important to note that different experimental protocols can be used to induce preload changes [6–8,12–17]. However, measuring the preload (that is, the length of cardiac fibers prior to contraction) is not really achievable *in vivo*, so there is an experimental need for a preload index, *i*.*e*., an indirect evaluation of the end-diastolic fibers length. Among the ventricular preload indices, we can cite the end-diastolic volume, the end-diastolic pressure, or the right atrial pressure. All these indices have however shown their limitations [10,18–21], and the search for an ideal preload index is still going on. Starling’s curves (like the one pictured in Fig 1) are a common visual representation of the FS mechanism found in the literature. Such curves are generally supposed to represent cardiac output variations upon change in preload, all other variables remaining constant. Since this procedure is hard to obtain experimentally, such curves are built as an ideal representation of the FS mechanism, rather than on experimental data, and thus they are mainly qualitative.

It is commonly admitted that the FS mechanism has a cellular origin, and that it arises from the length-dependent properties of the cardiac cells. More precisely, the FS mechanism is associated with length-dependent activation (LDA) [22–28] (see Fig 1). There is still a debate about the molecular mechanisms that are involved in LDA [27,28]. Recent experimental studies have demonstrated the existence of a “super-relaxed” state of myosin (or “OFF” state) [29,30]. Myosins are recruited from this super-relaxed state during activation to contribute to cross-bridge cycling and force-production. Once activation ends, most myosins come back to the OFF state. Some authors suggest that the recruitment from the OFF state could be force-dependent and may explain the increase in isometric force upon cell lengthening via a mechanosensing mechanism [31]. Hence, the giant protein titin, responsible for the passive elastic properties of the sarcomere, could be driving the length-dependent properties of force production [32]. The myosin-binding protein C, an important regulator of cardiac function, has also been proposed as key modulator of LDA [33]. Even if the LDA molecular mechanisms are complex and not fully elucidated yet, the generally admitted explanation regarding the cellular origins of the FS mechanism goes as follows [25,34]: if cardiac cell length increases, then the maximal produced force increases (signature of LDA), and so does the pressure and the amount of blood ejected by the ventricle. Here a delicate point must be highlighted: LDA is a cellular property that is mainly studied and described in *isometric* cellular experiments, where the cell length is fixed. On the other hand, the FS mechanism occurs *in vivo*, where cell length changes during the cardiac cycle. It has already been underlined that the length-tension relationship and the Starling Law could not directly be put in parallel [35–38]. It has also been shown that preload affects the velocity of shortening in isotonic experiments [39], and the timing and magnitude of contraction in auxotonic conditions [40,41]. The "positive effect of ejection" highlighted by Hunter [42] reveals how preload, among other parameters, may affect the whole dynamics of an ejecting beat [26,42,43]. These properties are rarely cited when discussing the cellular basis for the FS mechanism. The latter is somehow always associated with an increase in peak force following an increase in fiber length. The impact of preload on the whole dynamics of cardiac contraction is more rarely discussed.

It is especially important to investigate this question, as the FS mechanism is also assumed to be the basis for fluid therapy [20,44–46]. This therapy consists in intravenous administrations of fluid to a patient in order to restore their cardiac output. Indeed, an increase in circulating blood volume leads to an increase in preload and thus triggers the FS mechanism. If the patient is operating on the “ascending portion” of the FS curve, he is said to be “fluid-responsive” (see Fig 1), as a substantial stroke volume increase should be observed upon vascular filling.

As explained above, experimental difficulties may arise regarding the study of the FS mechanism. In particular, the connection between the FS mechanism and LDA or other cellular mechanisms is difficult to unravel experimentally, as three very different scales (cellular, ventricular and cardiovascular) are involved. Mathematical modeling of biological system is a growing field that helps complement the experimental data. Once validated, a model can overcome the experimental limitations and become a powerful instrument, either for clinical purposes, or for broadening physiological knowledge [47,48]. Regarding the FS mechanism, a multiscale model of the cardiovascular system (CVS) would help link the cellular properties (namely, LDA) to the ventricular and cardiovascular observations (namely, the stroke volume variations in response to preload variations), while providing a formal framework to integrate the experimental observations coming from the three scales. In this paper, such a multiscale model is described, and *in silico* protocols are proposed to analyze the FS mechanism in the left ventricle, its relationship with LDA and its relevance to fluid therapy.

In the following sections, our mathematical model of the CVS is described. Simulations that challenge the FS mechanism are implemented and the role of LDA is investigated. Then vascular filling simulations are carried out to further analyze the LDA function in fluid responsiveness.

## 2. Methods

**Fig 2:**
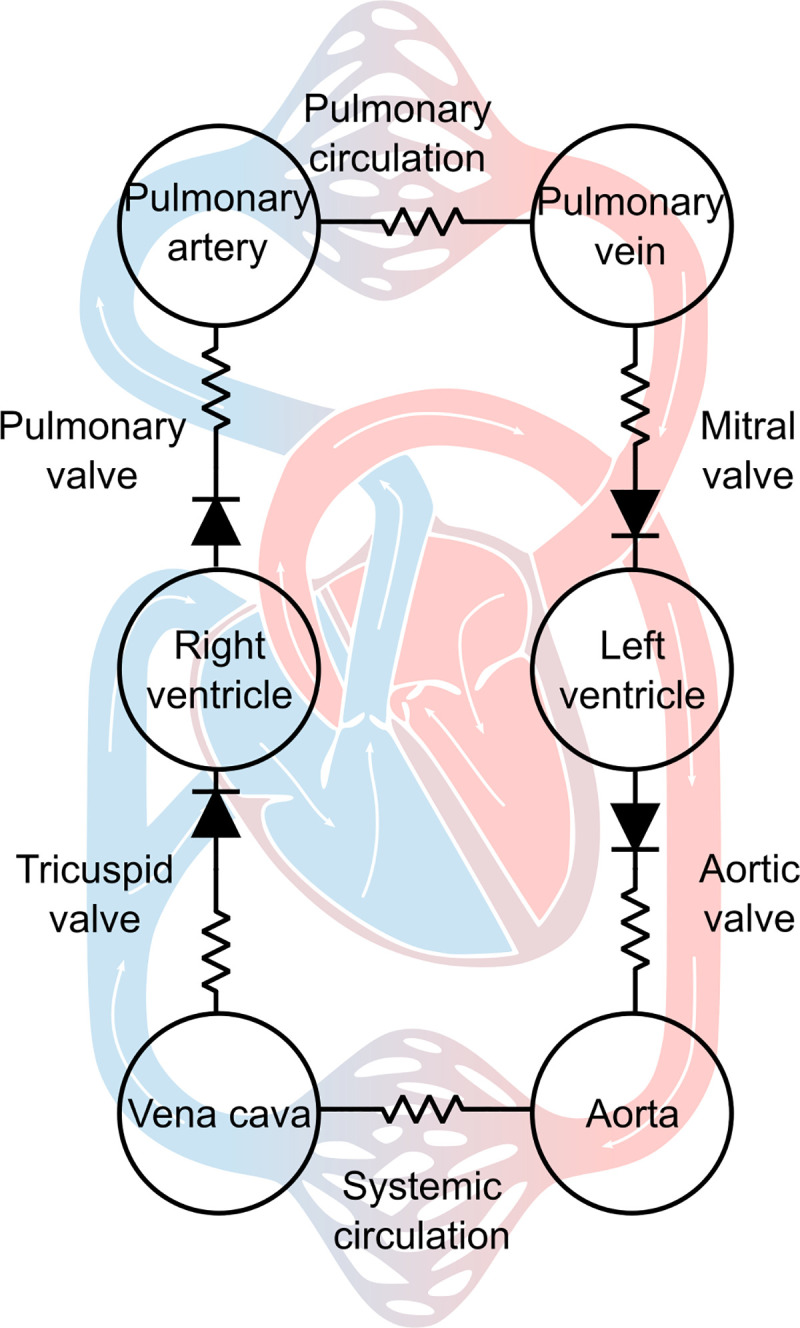
6-chamber lumped-parameter model of the cardiovascular system. Ventricular contraction is described at the cellular scale. The left and right ventricles are assimilated to spheres, and the force and length of a half-sarcomere are connected to the pressure and volume within the ventricular chambers.

Our multiscale model of the human cardiovascular system has already been described elsewhere, and the interested reader is referred to references [49,50] for all details and notations. The whole model equations and parameters can be found in the supplementary material (S1 Appendix), and it corresponds to the model presented in [50], where a few hemodynamical parameters were changed compared to [49] (see Table C from S1 Appendix). Briefly, the CVS is represented as a 6-chamber lumped-parameter model (see Fig 2). A simple thick sphere model for the left and right ventricles allows connecting the force and length produced by a half-sarcomere model [51,52] to the pressure and blood volume inside a spherical ventricle model [53].

**Fig 3:**
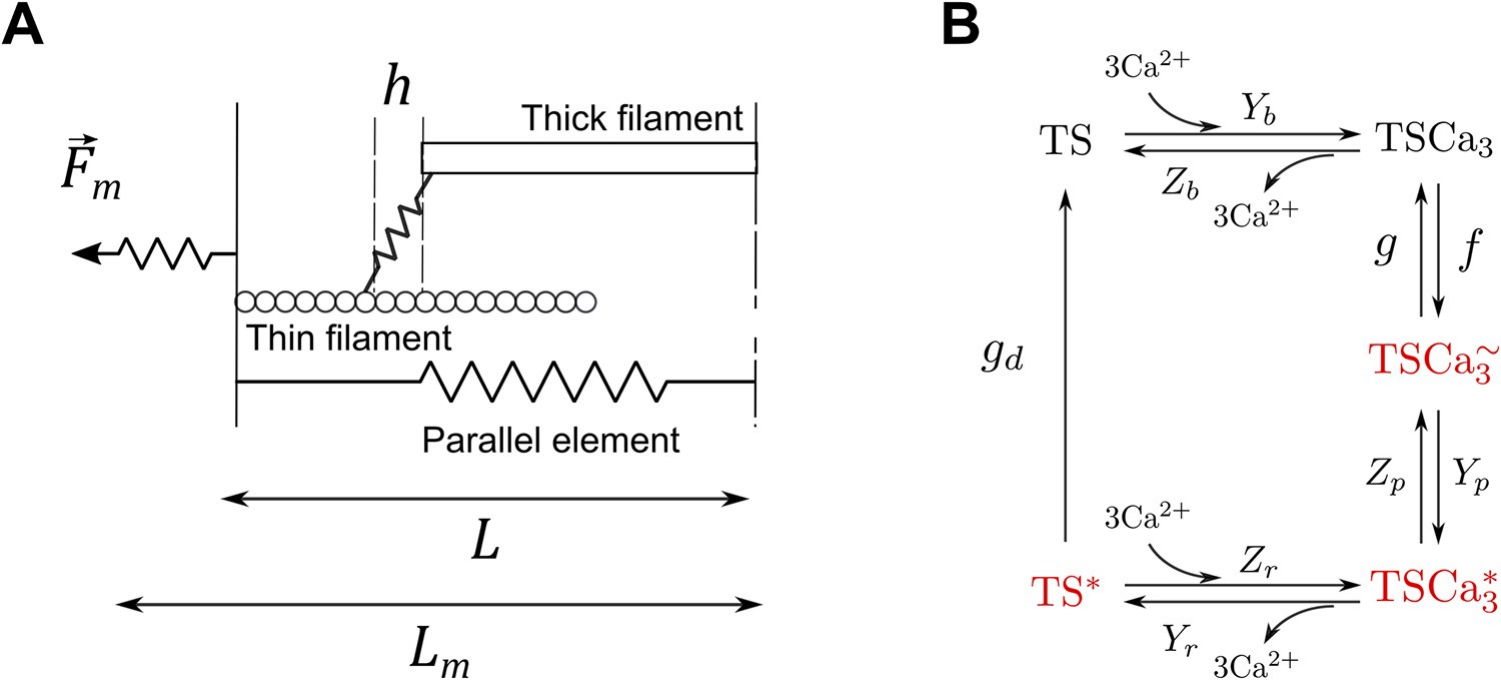
Half-sarcomere model. The half-sarcomere model describes the total force *F*_*m*_ produced by an active contractile element of length *L* (A). The crossbridges kinetic cycle is described with a 5-state model (B). The states highlighted in red correspond to attached crossbridges. Two of the rate constants, *f* and *g*_*d*_, are functions of the half-sarcomere length *L* (see Eqs 2–3).

Now more details are provided regarding LDA inside the model [49,51,52]. The biochemistry of the half-sarcomere contraction is described with a calcium kinetic model as shown in Fig 3. Here the basic unit for the crossbridge cycling model is chosen to be a troponin system (TS), defined as three adjacent troponin–tropomyosin regulatory units. The five states of the model are: free TS (TS), calcium bound to TS (TSCa_3_), calcium bound to TS with attached myosin heads (or crossbridges, CBs) in the weak state (TSCa_3_^~^), calcium bound to TS with attached CBs in the power state (TSCa_3_*), TS without calcium and with attached CBs in the power state (TS*). The active force is proportional to the TSCa_3_^~^, TSCa_3_* and TS* concentrations:

$$F=A_{w}[{\mathrm{TSCa}}_{3}^{∼}]h_{w}+A_{P}(\left[{\mathrm{TSCa}}_{3}^{*}]+[{\mathrm{TS}}^{*}\right])h_{p}$$ (1)

where *A*_*w*_ and *A*_*P*_ are constants, and *h*_*w*_ and *h*_*p*_ are the mean elongations of the weak and power CBs populations, respectively. Note that this force is normalized to the muscle cross-sectional area, and is thus expressed in Newton per unit area. In the figures and equations of this paper, cellular force is always expressed with units of stress.

Two of the rate constants from Fig 3, *f* and *g*_*d*_, are functions of the half-sarcomere length *L*:

$$f=Y_{a}\exp(-R{\left(L-L_{a}\right)}^{2})$$ (2)

$$g_{d}=Y_{d}\exp(-Y_{c}(L-L_{c}))$$ (3)

**Fig 4:**
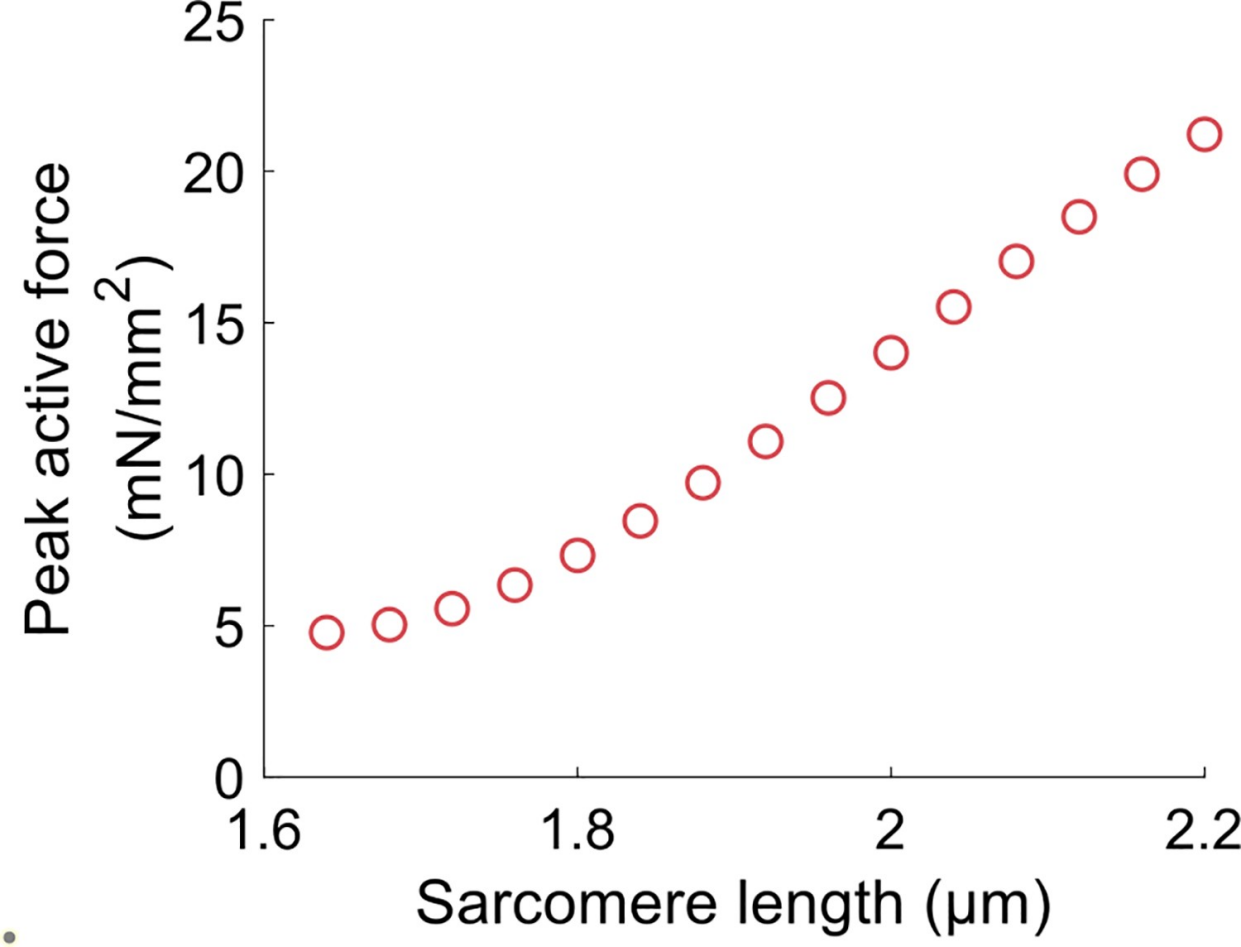
Length-tension relationship obtained with the half-sarcomere model. This curve is similar to Fig 11A from [51], as we use the same set of parameters for the crossbridge cycle rate constants. Force is normalized to the muscle cross-sectional area and is thus expressed in mN/mm^2^.

**Fig 5:**
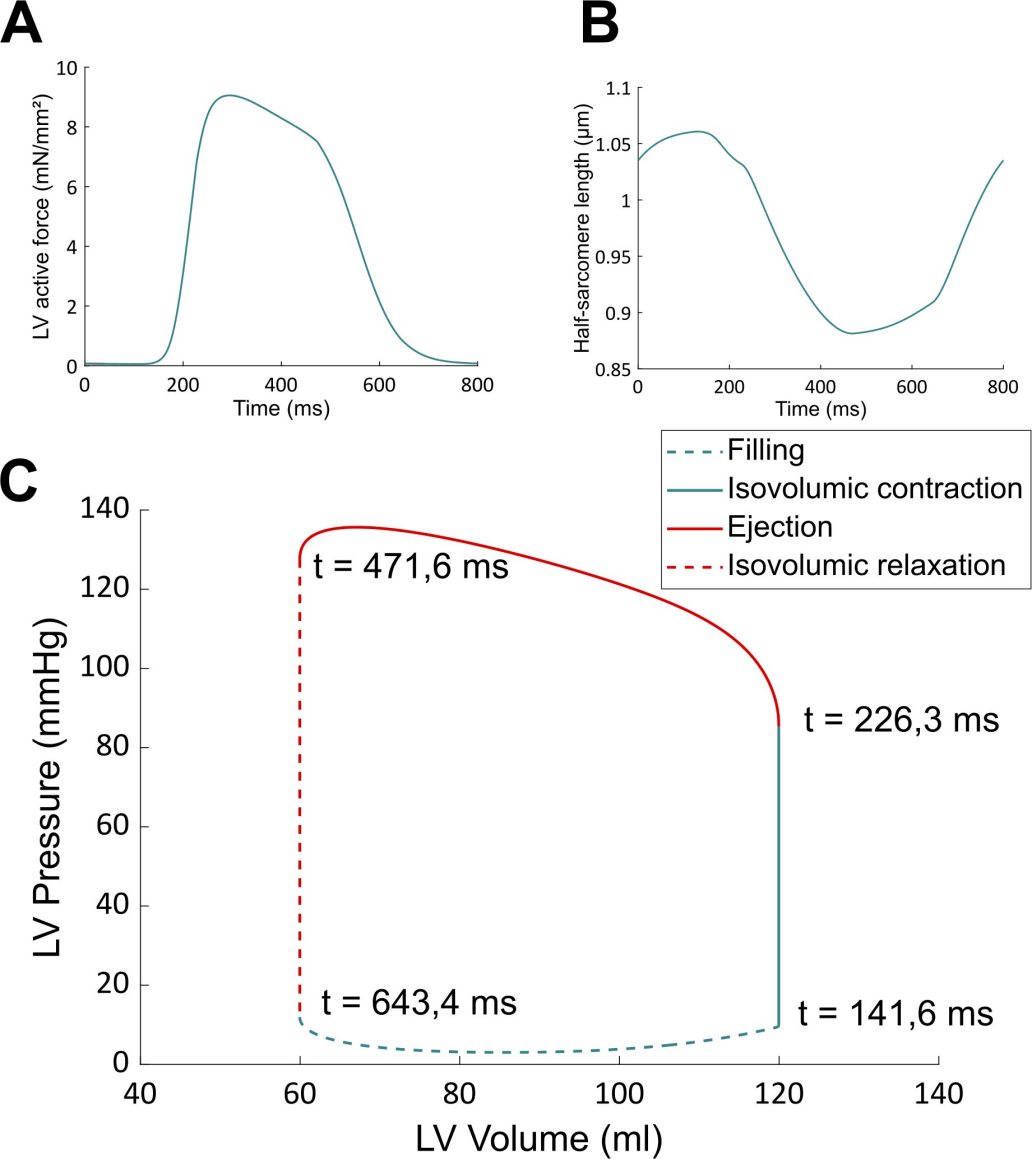
Baseline results obtained with the CVS model. These curves, that describe normal (healthy) hemodynamical conditions, are similar to Fig 5 from [49]. Time values on the PV loop indicate the beginning of each phase of the cardiac cycle for an 800 ms heartbeat.

Eq 2 describes a symmetrical CB attachment rate in the zone of overlap between thick and thin filaments. *L*_*a*_ is the optimal overlap length, *Y*_*a*_ is the maximal attachment rate (when *L* = *L*_*a*_), and *R* is the kurtosis of the curve [52]. Eq 3 describes an unsymmetrical and irreversible CB detachment rate. *L*_*c*_ determines the range of sarcomere length around which *g*_*d*_ varies, *Y*_*c*_ defines the rate of change of *g*_*d*_, and *Y*_*d*_ is the value of *g*_*d*_ when *L* = *L*_*c*_ [52]. This equation represents the lattice spacing effect on the rate of CB detachment: *g*_*d*_ decreases at larger length, where thick and thin filament are closer to each other. It is important to emphasize that these two equations actually define and describe LDA in our approach. Note also that this model does not allow to discriminate between potential contributors to LDA, but it is enough to reproduce a vast range of experimental results [51,52], such as the length-tension curve (shown in Fig 4), or the tension-pCa curve, which are two typical effects of LDA in cardiac muscle. We have used the same set of parameters for the crossbridge cycle rate constants than in [52] as a way to ensure that our model correctly reproduced the length-dependent properties of the cardiac muscle. In other words, this half-sarcomere contraction model is able to reproduce experimental data showing length-dependent activation. The model is also able to reproduce baseline behaviors of the CVS. See for instance the left ventricle pressure-volume loop from Fig 5. In this figure, the force and length of the half-sarcomere model are also represented. The hemodynamical parameters were fitted so that the hemodynamical variables (such as the mean ventricular pressure) and the half-sarcomere lengths range between physiologically relevant values (see details on the fitting procedure in S1 Appendix). The multiscale feature allows for a translation of cellular properties to the organ level. The results presented in Fig 5, among others published with this model [49], agree well with experimental observations and thus validate our modeling approach.

### Instantaneous increase in preload

Since the FS mechanism is actually a rapid response to an increase in preload, occurring within a single heartbeat, all other variables remaining constant, we propose the following protocol to study the FS mechanism *in silico*. We want to induce a rapid increase in left ventricular preload. We thus artificially increase mitral blood flow (which corresponds to the blood flow entering the left ventricle during the blood filling phase). This maneuver alters the blood volume distribution across the CVS (so that more blood enters the left ventricle), but the total circulating blood volume is kept constant (so no blood volume is artificially added to the circulation). This mitral blood flow increase is induced as a way to mimic a sudden increase in venous return, and thus preload, during ventricular filling (this preload increase can occur when legs are raised, for instance). Of course, the duration and amplitude of the mitral flow increase will dictate the end-diastolic volume. The longer/higher the flow increase, the greater the end-diastolic volume increase. To get a ~10% increase in end-diastolic volume, the mitral blood flow is artificially increased by 0,43 ml/ms during 60 ms in the left ventricular filling phase. Hence an increase in preload is induced at the beginning of the cardiac cycle and the variations in stroke volume are recorded within the same heartbeat. This constitutes the instantaneous increase in preload (IIP) protocol.

### The NO LDA model

In order to investigate the role of LDA in the response to an IIP protocol, we need to introduce a modified model, in addition to our multiscale model of the CVS presented above. As a first step in introducing this modified model, which will be referred to as the “NO LDA model”, let us remind that the FS mechanism is an *adaptation* of the CVS behavior to a change in preload and that our purpose is to examine the role of LDA on this *adaptation*. It is also important to emphasize that the sarcomere *length* appears in many places in the equations that govern the CVS (see all these equations in S1 Appendix) and this variable thus plays a quite complex role in the behavior of the system. Then let us emphasize again that the standard and usual definition of LDA in the literature only refers to isometric (and thus stationary) contractions, for which the sarcomere length is constant over time. In particular, let us strongly insist that since LDA is defined in a stationary context, this concept is in essence not linked with the contraction velocity or rate of change of the sarcomere length. LDA refers to the dependence of the crossbridge cycling process with respect to the *value* of the sarcomere length, and independently of the influence of the contraction velocity, or of any other effects.

In that context, the analysis of the influence of LDA on the FS mechanism consists in studying how the modified *values* of the sarcomere length induced by an IIP protocol could influence the behavior of the crossbridge cycling, and consequently the behavior of the whole CVS. Of course, the adaptation of the system to the IIP-induced changes of the *values* of the sarcomere length is accounted for in our original multiscale model through the length (value)-dependence of the crossbridge cycling process, *i*.*e*., through the *L*-dependence of *f* and *g*_*d*_. To assess the importance of LDA in the *adaptation* of the CVS in an IIP protocol, we thus need to compare the response of our multiscale model to that protocol to a fictive evolution for which LDA would be “switched off” during the adaptation. In that fictive evolution, the crossbridge cycling process should not be affected by the modified values of the sarcomere length that are imposed by the IIP protocol. This amounts to preventing the *L*-values changes with respect to the pre-IIP situation to influence the crossbridge cycling process. This is also equivalent to considering that the values of the sarcomere length that must be considered to describe the crossbridge cycling process during the IIP protocol are those that would have existed without this IIP protocol, *i*.*e*., the pre-IIP values, or also the baseline values, as described by the original multiscale model.

To build the so-called NO LDA model, whose purpose is to describe the fictive evolution introduced above, the length (value)-dependence of *f* and *g*_*d*_ must thus modified by replacing in Eqs 2–3 the true length *L* by its baseline (BL) counterpart. This quantity will be denoted *L*_*BL*_ and it must be considered as an “input function” in the model. One thus gets for the NO LDA model:

$$f=Y_{a}\exp(-R{\left(L_{BL}-L_{a}\right)}^{2})$$ (4)

$$g_{d}=Y_{d}\exp(-Y_{c}(L_{BL}-L_{c}))$$ (5)

**Fig 6:**
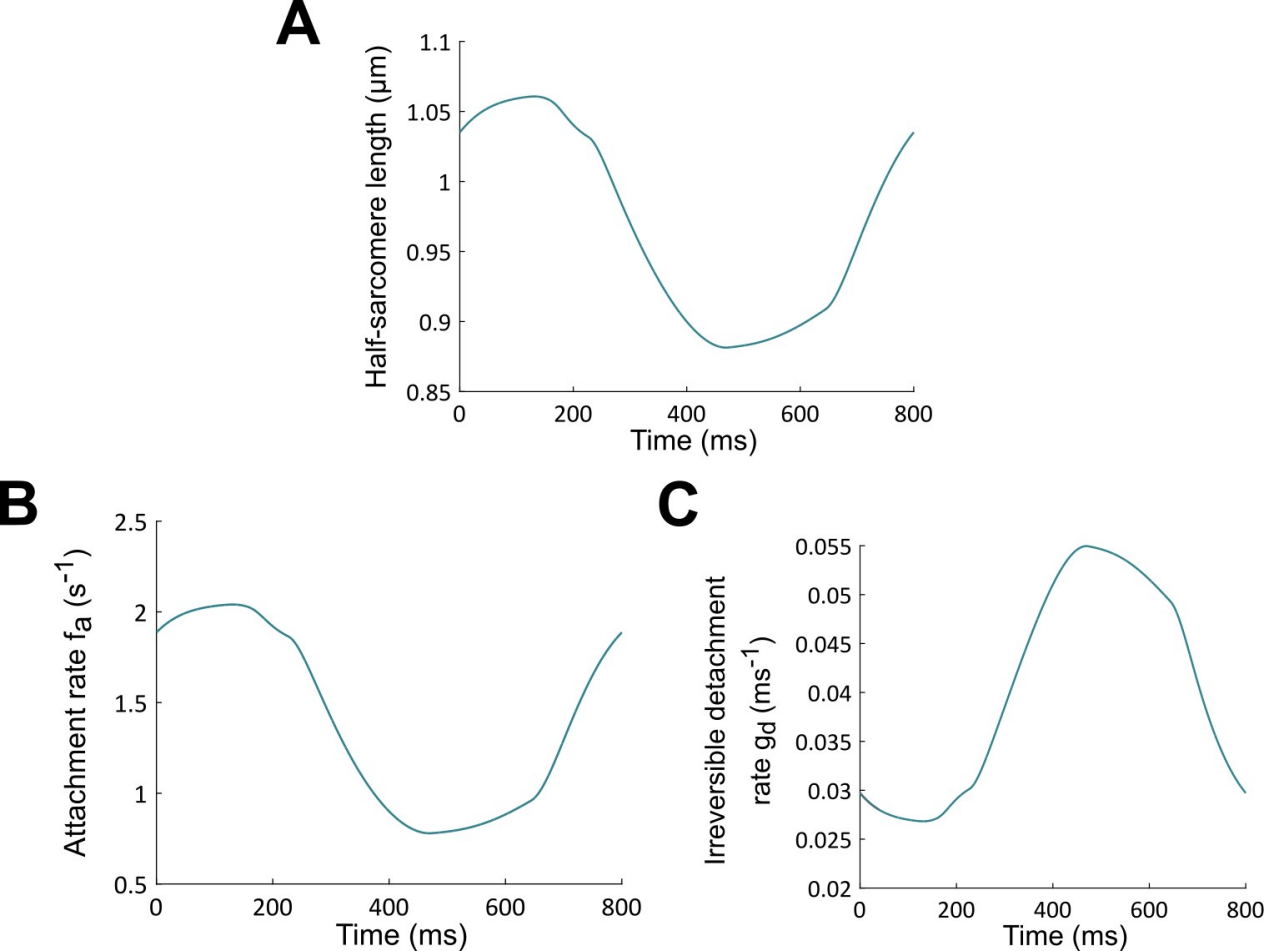
Half-sarcomere length *L*_*BL*_ during a baseline heartbeat (normal hemodynamics conditions) (A). Associated attachment rate *f* (B) and irreversible detachment rate *g*_*d*_ (C). These rates are used as input functions for the NO LDA model, in such a way that panels B and C also correspond to the attachment rate *f* and irreversible detachment rate *g*_*d*_ for the NO LDA case.

The “input function” *L*_*BL*_ and the associated rates *f* and *g*_*d*_, which correspond to the results of Fig 5, are plotted in Fig 6. In the modelling of the IIP protocol with the NO LDA model, the length dependence of crossbridge cycling is thus “frozen” to its BL behavior, which also means that the *adaptation* of the crossbridge cycling process to the IIP-induced changes of the *values* of the sarcomere length is actually switched off. The true length of the sarcomere is absent in the modeling of the crossbridge cycling process, which truly means that no LDA is present in the NO LDA model. Let us also emphasize that the velocity-dependence of CBs kinetics and mean CBs elongations is preserved in the NO LDA model (see S2 Appendix, Eqs 2–3 and 6), so there is still some dependency with respect to the *time evolution* of the sarcomere length in the description of the adaptation of the system (this is usually referred to as the”velocity-dependence” of contraction). This dependency on the length variation rate is responsible for important myocardium properties such as shortening deactivation [54,55]. However, as explained above, this effect is not related with the standard notion of LDA and must thus be kept in our NO LDA model.

The question we examine below is whether, and to what extent, this NO LDA model positively responds to a preload increase in the (auxotonic) contraction defining a heartbeat. Obviously, when no increase in preload is imposed on the left ventricle, the NO LDA model leads to the baseline results presented in Fig 5, but if preload (or any other factor) is modified, the results are expected to differ.

### Vascular filling

In the CVS model, the stressed blood volume is an adjusted parameter that characterizes the total amount of circulating blood volume responsible for a non-zero pressure inside the system. To mimic the increase in circulating blood volume during fluid therapy, the stressed blood volume is increased, and we let the system stabilize to reach its new steady state. Simulations where stressed blood volume is decreased are also performed to mimic vascular emptying. Since vascular filling induces adaptations with respect to an initially stabilized situation, the NO LDA model can also be used in the present situation to evaluate the LDA contribution to fluid responsiveness. Note that, in contrast to the analysis of the FS mechanism for which the instantaneous response is observed, here we wait for the system to stabilize. This is because vascular filling therapy aims at achieving a better steady state following fluid injections.

## 3. Results

All simulations whose results are discussed below were performed in MATLAB (The MathWorks, Inc., Natick, MA, USA). The computer code is freely available under the GNU General Public License and can be downloaded at https://github.com/Sarah-Kst/Mutli_CVS_model.

### The Frank-Starling mechanism

Within the modeling framework described in the previous section, we can now challenge the nature of the relationship between LDA and the FS mechanism. More precisely, we can investigate the hypothesis of a connection between maximal force and stroke volume.

**Fig 7:**
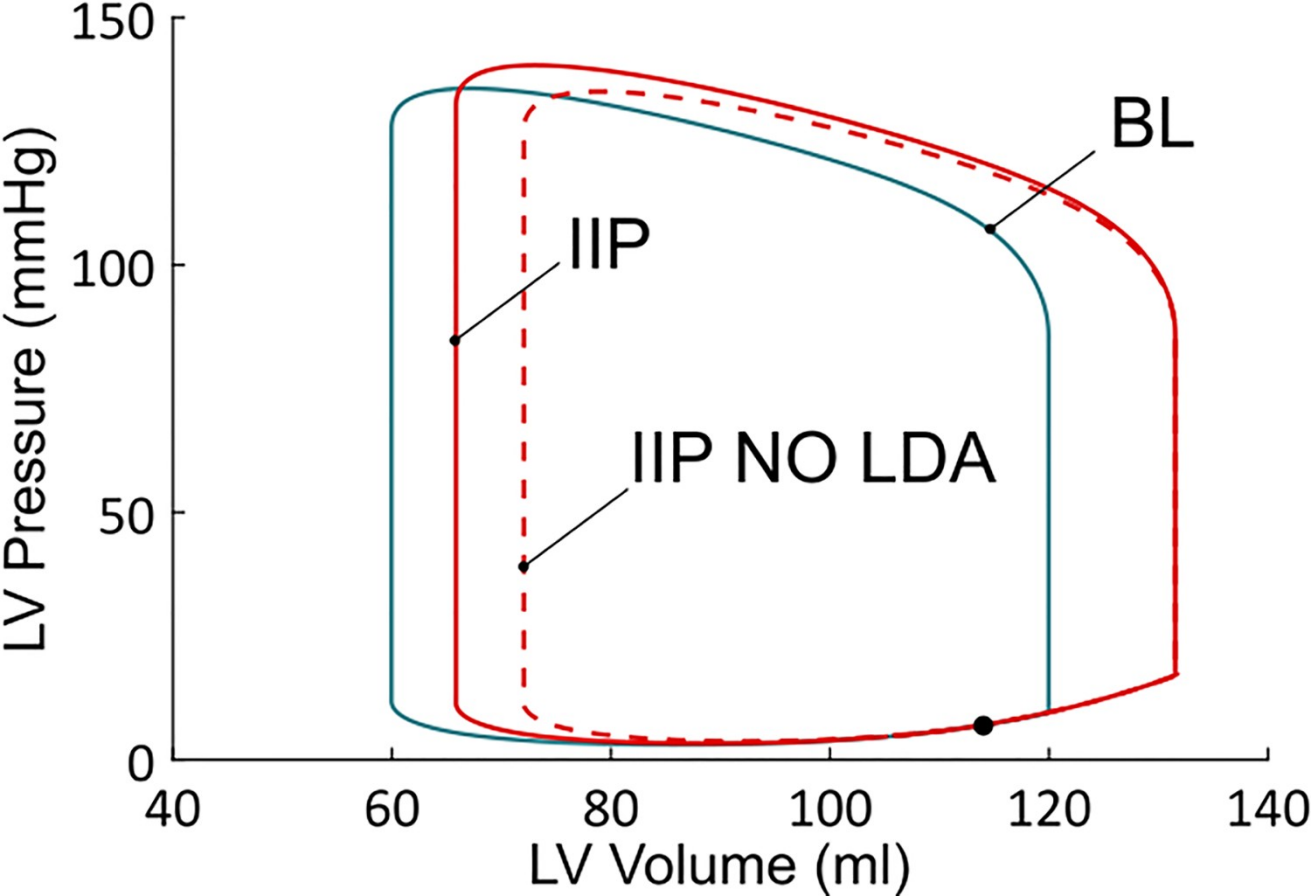
Left ventricular PV loops from the BL, IIP and IIP NO LDA simulations. IIP starts during ventricular filling (black dot). PV loop for the baseline case (blue) corresponds to a stabilized behavior (each heartbeat leads to that same PV loop). On the other hand, the two PV loops for the IIP protocols (red dashed or solid lines) correspond to a transient response to a preload change. The behavior is not stabilized yet, as this would take several more heartbeats to return to the baseline state. Those PV loops are thus not closed, but this it cannot be seen in the picture.

Pressure-volume (PV) loops obtained from the BL case and from the IIP protocol are shown in Fig 7. We observe first that there is an increase in end-diastolic volume upon IIP. This means that preload has increased. Stroke volume from the IIP beat has also increased compared to the BL case (the PV loop is larger, see also Table 1). There is also an increase in maximal force and pressure, as presented in Table 1. But the question that is addressed here is whether LDA is the underlying mechanism responsible for the stroke volume increase. To answer this, the IIP protocol is also performed with the NO LDA model. As can be seen from the NO LDA PV loop in Fig 7, it is clear that stroke volume decreases when LDA is frozen (with respect to the LDA model). Actually, stroke volume with the NO LDA model is very close to the BL stroke volume, which means that the CVS did not respond to the increase in preload.

**Table 1:** Quantitative comparison of the BL, IIP and IIP NO LDA simulations. See text for detailed explanations.

	End-diastolic Volume (ml)	Stroke volume (ml)	Maximal Active force (mN/mm^2^)	Maximal Pressure (mmHg)	Ejection duration (ms)
BL	119,96	60	9,05	135,65	245,28
IIP	131,53 ***(+ 9*,*65%)***	65,68 ***(+ 9*,*47%)***	9,69 ***(+ 7*,*07%)***	140,37 ***(+ 3*,*48%)***	257,44 ***(+ 4*,*96%)***
IIP NO LDA	131,54 ***(+ 9*,*66%)***	59,50 ***(- 0*,*84%)***	9,54 ***(+ 5*,*34%)***	135,06 ***(- 0*,*44%)***	246,40 ***(+ 0*,*96%)***

In Table 1, stroke volume, maximal produced force and pressure are presented and compared between BL and IIP, with and without LDA. A surprising result is that the maximal active force still increases upon IIP when LDA is off (+ 5,34% compared to the BL case, but - 1,62% compared to the LDA active situation), while stroke volume stays roughly the same as for the BL case (- 0,84% compared to the BL case, and - 9,41% compared to the LDA active situation). This increase in maximal force is due to an increase in the number of force-generating CBs compared to the BL case, as well as an increase in their elongation (see Eq 1). This happens even in the absence of LDA because the CBs concentrations and CBs elongations are still dependent on the time evolution of the sarcomere length variations in the cell model. A more complete explanation on the increase in maximal force observed in both situations (LDA active and LDA off) is provided in the supplementary material (S2 Appendix). This result suggests that the stroke volume increase following IIP when LDA is present is not directly mediated through an increase in generating force capacity. In other words, the presence of LDA does not involve an increase in maximal force which would be explicitly responsible for an increase in stroke volume. Maximal pressure, on the other hand, did not significantly change in the IIP protocol once LDA was turned off (- 0,44% with respect to the BL case, and - 3,78% compared to the LDA active situation). It should be emphasized that pressure does not only depend on force but also on ventricular volume. More precisely, pressure depends on total produced force (*F*_*m*_), total sarcomere length (*L*_*m*_) and ventricular dimensions (*r*_*out*_ and *r*_*in*_, resp. the external and the internal radius) according to the following equation (see S1 Appendix):

$$P_{lv}=7.5\;F_{m}\frac{L_{m}}{L_{r}}{\left[(\frac{r_{out}}{r_{int}}\right)}^{2}-1)]+\lambda (V_{lv}-V_{o})$$ (6)

We see here that the nature of the relationship between force and pressure is complex, and it depends on the geometry of the ventricular chamber. Any change in sarcomere length *L*_*m*_ upon IIP will influence cellular force production, which will influence ventricular pressure development. This in turn will influence blood ejection and ventricular dimensions, which will change *L*_*m*_. The three variables (sarcomere length, force, and pressure) are thus dynamically interconnected, and force and pressure should not be seen as interchangeable variables. A more detailed analysis of the length, CBs rates, CBs concentrations, force, and pressure evolutions upon IIP with and without LDA is provided in S2 Appendix for the interested reader.

**Fig 8:**
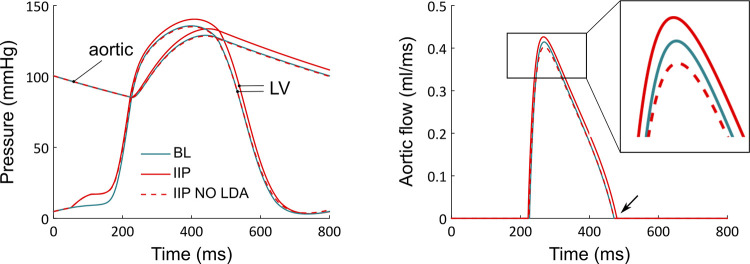
A. Ventricular and aortic pressures during a BL (blue), IIP (red), and IIP NO LDA (dashed red) simulation protocol. B. Corresponding aortic flows. The aortic flow amplitude (inset) and ejection duration (black arrow) increase for the IIP protocol compared to the BL case, but not for the IIP NO LDA protocol.

We can investigate more carefully the stroke volume variation upon preload increase by looking at flows instead of pressures. Indeed, stroke volume is given by:

$$SV=\int_{\begin{array}{l} \mathrm{ejection}\\ \mathrm{period} \end{array}}Q_{ao}(t)dt$$ (7)

where $Q_{ao}=\frac{P_{lv}-P_{ao}}{R_{vao}}$ is the aortic flow. In this expression, *R*_*vao*_ is the aortic valve resistance, *P*_*lv*_ is the left ventricular pressure and *P*_*ao*_ is the aortic pressure. The latter is a major component of afterload, which opposes blood ejection. The time evolutions of *P*_*lv*_ and *P*_*ao*_ are shown in Fig 8A. The aortic valve opens when *P*_*lv*_ gets greater than *P*_*ao*_, and blood ejection begins. Upon IIP, the aortic valve opens roughly at the same time and for the same value of *P*_*ao*_ than for the BL case, but *P*_*lv*_ reaches higher values during the ejection. An increase in stroke volume is thus expected. But, as more blood is indeed ejected through the aorta, *P*_*ao*_ increases and further opposes blood ejection. This participates in a reduction of stroke volume. However, the net result is actually an increase in stroke volume, as can be seen from the aortic flow shown in Fig 8B (stroke volume is basically given by the area under the aortic flow). Indeed, we observe that not only does the *Q*_*ao*_ amplitude increase upon IIP, but the ejection period also lasts longer (black arrow). The ejection ends when the aortic valve closes, which occurs once *P*_*lv*_ gets lower than *P*_*ao*_. An increased ejection duration thus means that the preload variations modified the *whole dynamics* of left ventricular and aortic pressures developments. This point will be developed further in the discussion section. From Table 1, we see that a greater developed pressure is obtained (+ 3,48%) and the ejection duration lasts longer (+ 4,96%) upon IIP. Both effects finally lead to an increased stroke volume upon IIP.

Once LDA is switched off, however, the aortic flow does not reach greater values (the maximum even decreases), and both the maximal pressure and the ejection duration are almost unaffected with respect to BL by the preload increase (respectively - 0,44% and + 0,96%). This leads to a negligible stroke volume variation (- 0,84%), compared to the IIP with LDA case (+ 9,47%). We see that LDA is required to obtain a stroke volume increase upon IIP.

**Fig 9:**
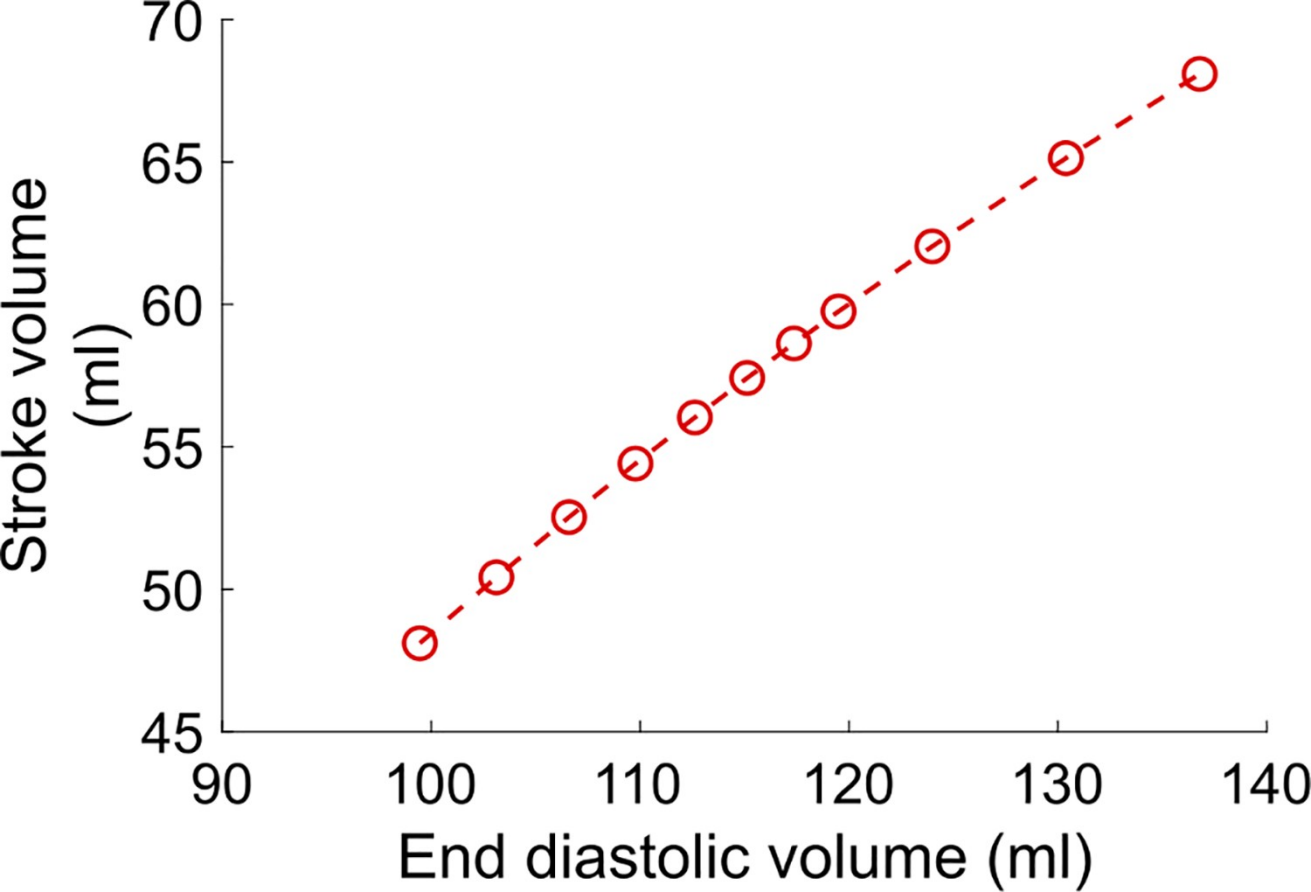
Frank-Starling curve obtained with the CVS model.

We can also obtain an FS curve similar to Fig 1 by applying different *Q*_*mt*_ increases in the IIP protocol. This is shown in Fig 9, where stroke volume is reported as a function of preload (preload is calculated as the maximal half-sarcomere length). Obtaining an FS curve experimentally is very challenging for a few reasons. First of all, the length of cardiac fibers is not measurable *in vivo*, and preload indices are used instead. Second, changing the preload while keeping all the other variables constant is hardly manageable in practice. With our model, however, we can easily build an FS curve which is consistent with the FS mechanism definition, by performing IIP protocols for different *Q*_*mt*_ increase (or decrease) values. We see that stroke volume is an increasing, and almost linear function of preload, and we do not observe the commonly assumed curvilinear shape or the plateau phase for high preloads (see Fig 1). One possible explanation for this is that the shape of the experimental FS curves actually depends on the chosen preload index [14]. For instance, a saturating portion of the curve may be expected if the end-diastolic pressure is chosen as the preload index, given the non-linear compliance of the ventricle. The absence of a plateau phase may also be related to the model limitations (see [49] and the Discussion section below). The important thing to note here is that, when drawing a FS curve, it is essential to explicitly state which preload-increase protocol is applied, and which preload index is measured.

### Vascular filling

**Fig 10:**
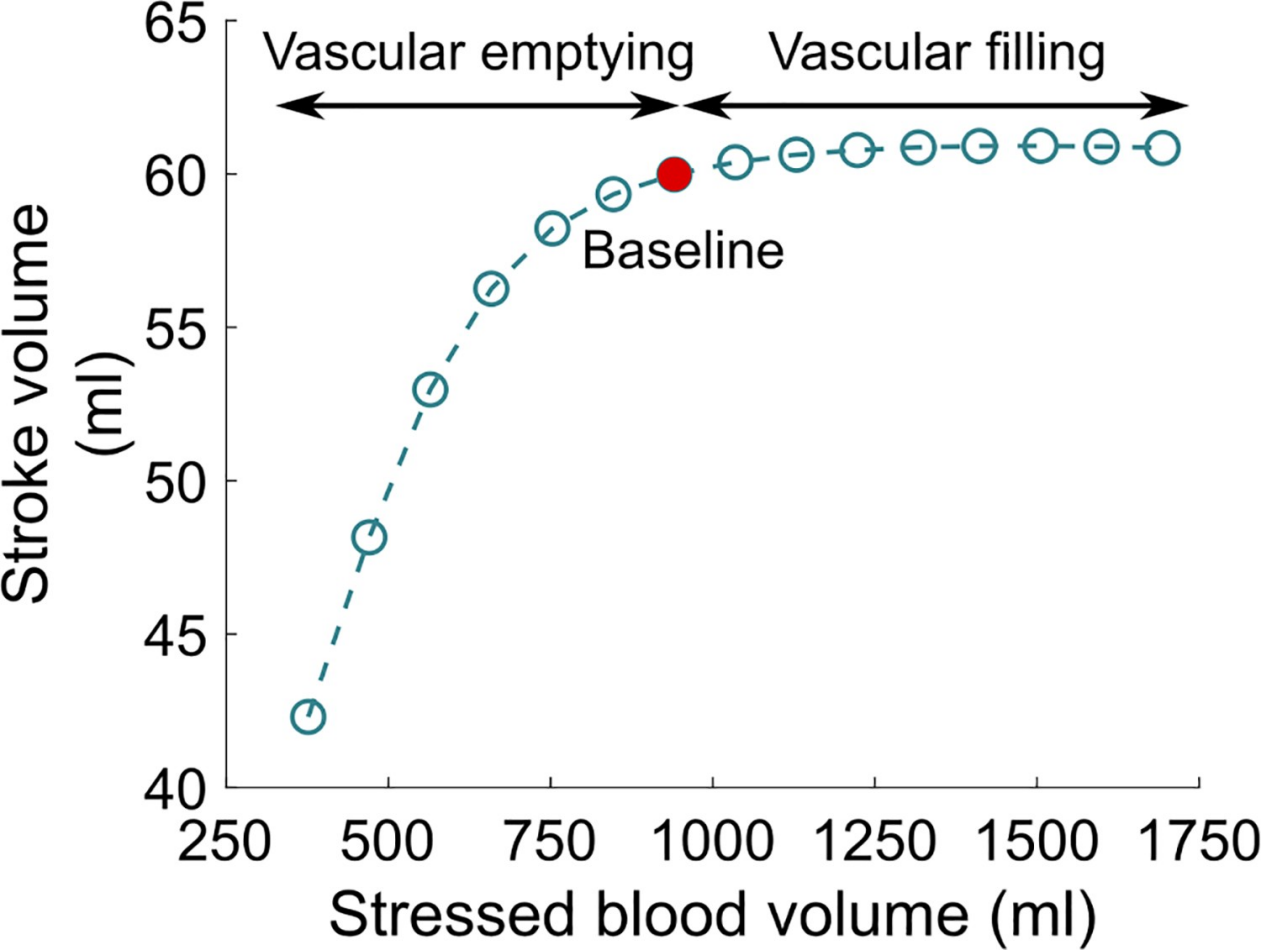
Vascular filling simulations. Stroke volume increases with filling, up to a saturating plateau. The red filled circle corresponds to the BL case (normal hemodynamics conditions).

**Fig 11:**
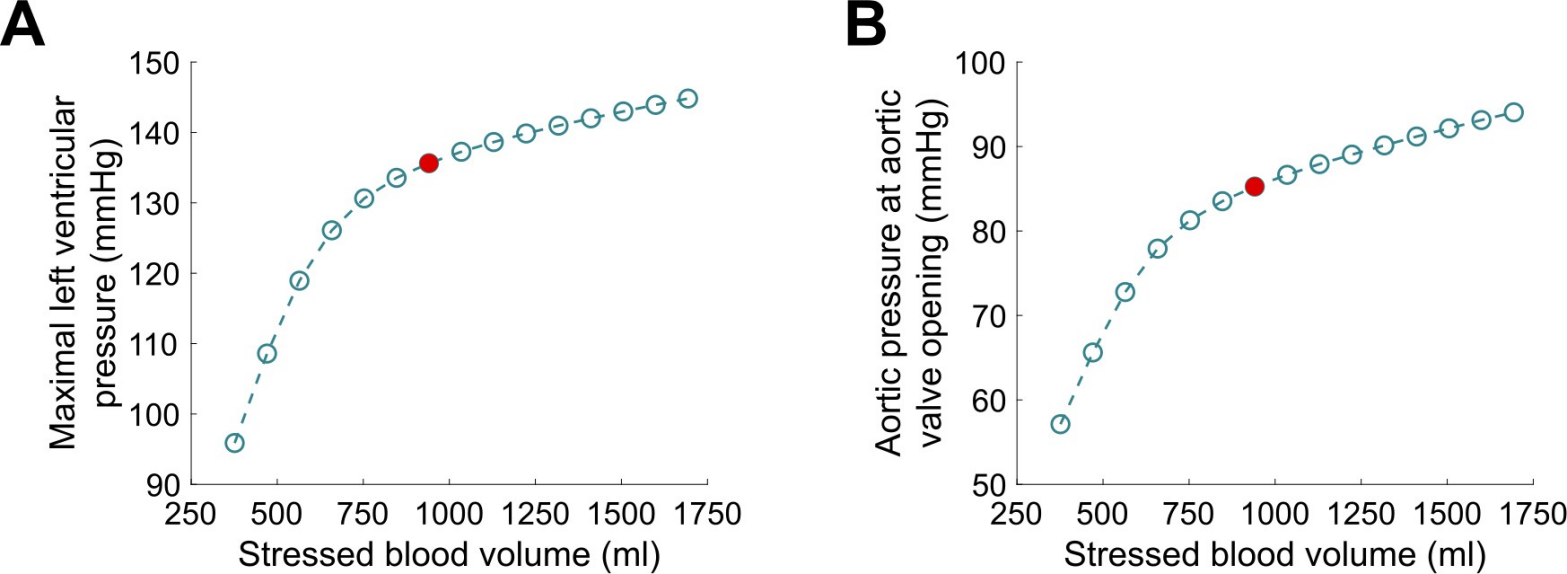
A. Maximal left ventricular pressure during vascular filling simulations. B. Aortic pressure at valve opening during vascular filling simulations. The latter is used to quantify the afterload.

**Fig 12:**
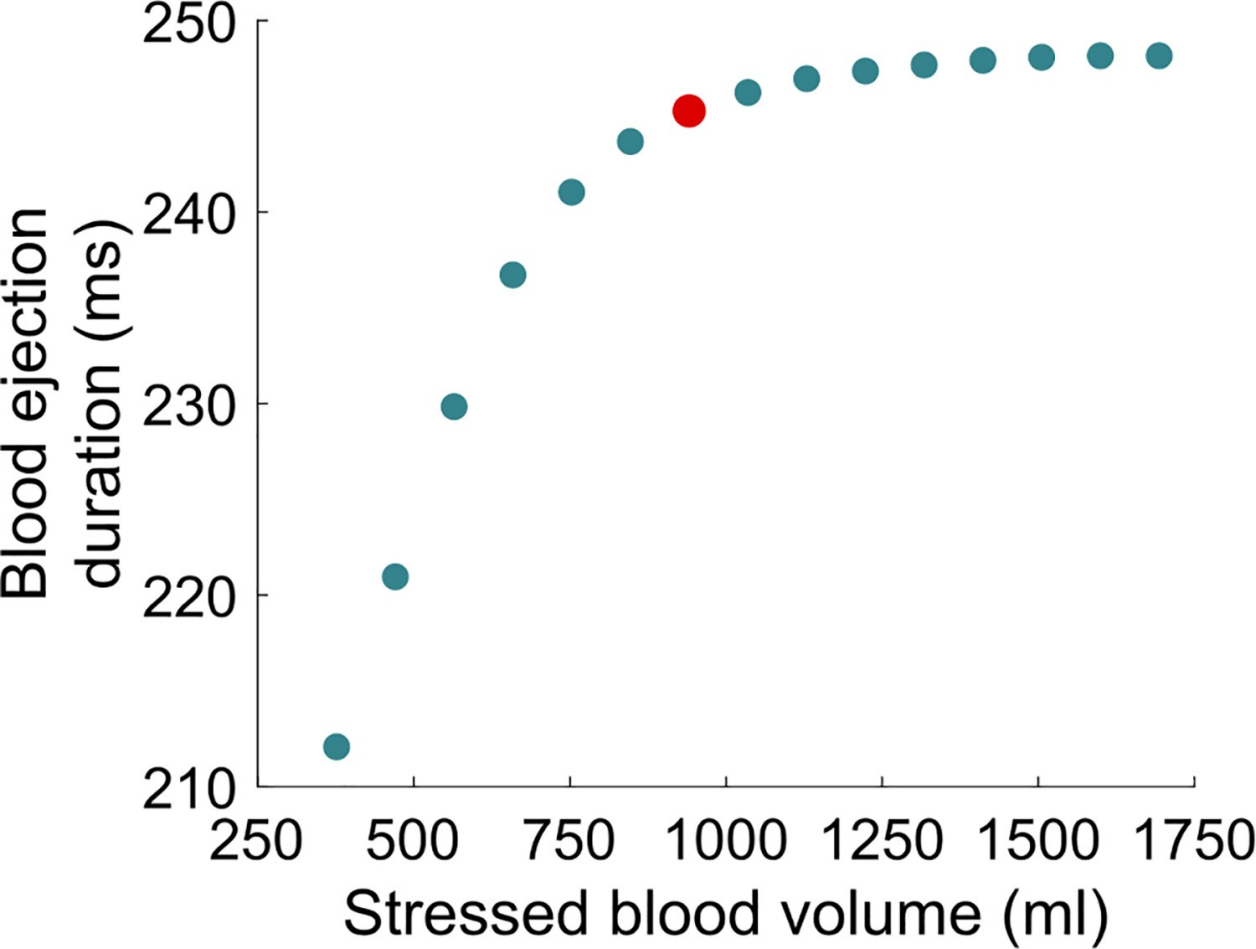
Blood ejection duration during vascular filling simulations.

**Fig 13:**
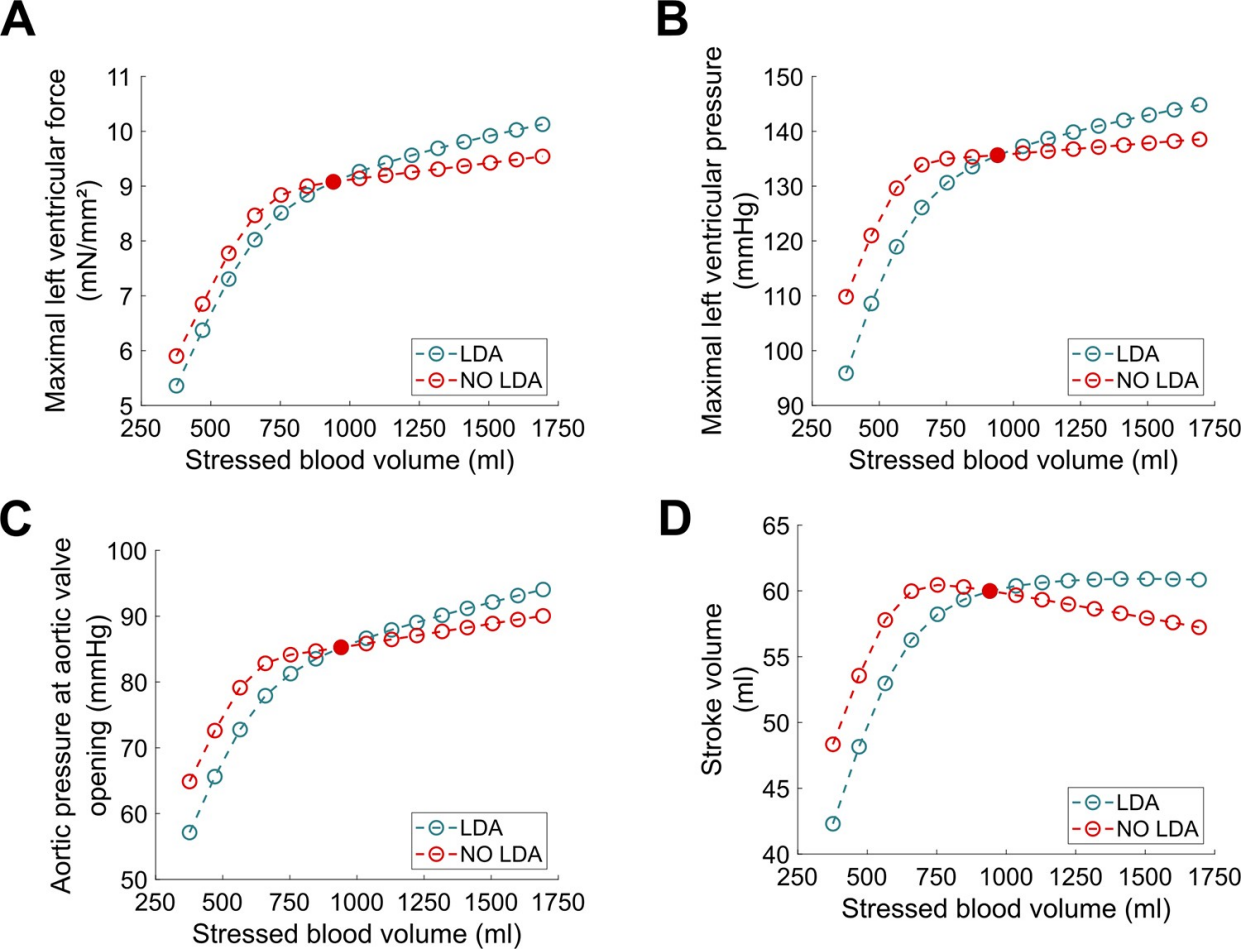
A. Maximal left ventricular force with (blue curve) and without (red curve) LDA. B. Maximal left ventricular pressure with (blue curve) and without (red curve) LDA. C. Afterload with (blue curve) and without (red curve) LDA. D. Stroke volume with (blue curve) and without (red curve) LDA.

The results describing the vascular filling simulations are presented in Figs 10–13. The red filled circle on the curves always corresponds to the baseline situation introduced above (normal hemodynamics conditions as shown in Fig 5). Points located at the right side of the baseline red filled circle correspond to vascular filling. Points located at the left side correspond to a vascular “emptying”, *e*.*g*., as a result of a hemorrhage. In Fig 10, we see that stroke volume increases with filling, up to a certain limit where it saturates. Since we know that the competition between *P*_*lv*_ and *P*_*ao*_ actually dictates the stroke volume (see Eq 7), maximal *P*_*lv*_ is shown as a function of stressed blood volume in Fig 11A. We see that it increases with filling but does not reach a plateau phase. *P*_*ao*_ at aortic valve opening, which we use to quantify afterload, is shown in Fig 11B. It also increases with filling, without any plateau phase either. Finally, the ejection duration is plotted in Fig 12, and a saturation is observed for high stressed blood volume values. This means that, even if the left ventricular pressure development is enhanced with vascular filling, for high stressed blood volume values it is counterbalanced by the increase in afterload. This leads to a saturated ejection duration and a saturated stroke volume.

Vascular filling simulations performed with the NO LDA model show that LDA is required to get a positive response, *i*.*e*., a stroke volume increase, to filling for high stressed blood volume values. Indeed, maximal produced force and pressure increase more marginally with filling once LDA is turned off (see Fig 13A and 13B). This is not enough to counterbalance the increase in afterload (Fig 13C), and thus stroke volume decreases for high stressed blood volume values (see Fig 13D).

## 4. Discussion

The FS mechanism is a rapid response induced by an acute increase in preload, which results in an increased stroke volume. The common given explanation of this phenomenon is that, as preload increases, LDA is triggered and the maximal produced force increases, and so does the stroke volume. It is important to remember that LDA is essentially highlighted and defined in *isometric* cellular experiments, while cardiac fibers actually undergo auxotonic contractions in a beating heart. This is why we developed a model where the dependence on the length values is switched off, while the dependence on the length variations (velocity-dependence) is unchanged. The aim was to study the adaptation to preload variations of a system where the crossbridge cycle does not depend on the true value of the sarcomere length anymore (= NO LDA), compared to a system with LDA. Then, to investigate the nature of the relationship between LDA and the FS mechanism, which is hard to study experimentally, we proposed an Instantaneous Increase in Preload protocol performed on a multiscale model of the human CVS. We showed that:

1. As preload increases, both the maximal force and pressure increase, but not to the same extent.
2. The maximal force is not a reliable predictor for stroke volume variations upon IIP. Instead of force, pressure should be considered. Those concepts are not interchangeable, as developed pressure also depends on ventricular geometry.
3. LDA is required to obtain an increase in stroke volume upon IIP.
4. An altered preload sets new initial conditions at the beginning of contraction. As a consequence, the whole dynamics of force and pressure development is altered. The aortic flow reaches a higher amplitude, and the ejection duration is extended. This leads to an increased stroke volume.

We see here that three different scales are involved in the cardiovascular system response to preload variations: cellular, ventricular, and cardiovascular. We can conclude from these observations that the FS mechanism is a multiscale and dynamical LDA-driven response to a change in preload. This response does not only manifest as an increase in produced cellular force, but also as an altered dynamic of contraction. We should note that this increased "ejecting-capacity" of the ventricle upon preload increase implies a secondary increase in afterload. Aortic pressure indeed increases upon IIP (see Fig 8A). This partially counterbalances the stroke volume increase. In other words, an increase in blood volume ejection capacity actually increases the aortic pressure, as more blood is ejected into the aorta. The afterload thus increases and tends to lower the expected increase in stroke volume.

The schematic FS curve from Fig 1 is portrayed in many textbooks and in the literature, but the underlying explanation can be vague or even incorrect. First, the general context is not always clarified. The FS mechanism is a rapid response that occurs on a beat-to-beat basis when only the preload is varied, all other variables remaining constant. Second, our results show that the increase in maximal force does not provide a sufficient and complete explanation of the stroke volume increase. Finally, there is a secondary increase in afterload following preload alteration. Since all these observations are rarely taken into account regarding the FS mechanism definition, inconsistencies can be found regarding the proposed FS curves. On the other hand, obtaining such curves experimentally is very challenging. With our model, we could build an FS curve consistent with the definition of the FS mechanism, by performing several IIP protocols with different mitral flow increased (or decreased) values. We show that stroke volume is an increasing and almost linear function of preload. As noted by Glower *et al*. [14], the shape of the experimental FS curve can go from linear to curvilinear depending on the chosen preload index. It is worth noticing once again that all the proposed preload indices (end-diastolic volume or pressure, atrial pressure) have their limitations, and the cardiac fibers length prior to contraction is a challenging measure to obtain experimentally.

The FS mechanism is often considered as a strong basis for fluid therapy. The general idea is to extend the transitory FS mechanism to a stabilized behavior. From a clinical perspective, the aim of vascular filling is to increase and stabilize the preload so that the stroke volume increase can be maintained, and the cardiac output improved. Our analysis shows that it is delicate to associate the transitory FS response with a stabilized increased cardiac output. The misunderstanding may arise from the fact that both the FS mechanism and vascular filling therapy are based on the length-dependent properties of cardiac cells. As we have shown in this study, LDA indeed underlies both phenomena, but this does not mean that the FS mechanism is linked to the vascular filling response. We propose to introduce instead the concept of *length-dependent fluid response* (LDFR), which we define as the mechanism underlying the motivations for vascular filling therapy: as circulating blood volume increases, preload increases and a stabilized substantial increase in stroke volume is expected if the patient is fluid-responsive.

Upon vascular filling, there is a competition between an increased produced pressure and an increased resistance to blood ejection. Our results indicate that for high stressed blood volume values, the LDA-driven increase in pressure development is not strong enough to counterbalance the afterload increase. This leads to a saturating ejection duration as well. As a global result, the stroke volume follows the same tendency and saturates for high stressed blood volume values. LDFR is thus a multiscale phenomenon where cellular, cardiac, and hemodynamic variables determine the global CVS behavior. Like for the FS mechanism, we observe a complex, multiscale response to preload variations (although the relative contribution of each scale is probably different than for the FS mechanism). Our results suggest that attention should not be drawn only to the preload during fluid therapy, because the afterload is also altered and definitely involved in stroke volume changes. In these simulations, we defined afterload as the aortic pressure at aortic valve opening in order to quantify it. But the resistance to ejection occurs throughout the whole ejection period, and this resistance increases with filling. Our results indicate that this leads to a saturating limit for vascular filling procedures. It is important to emphasize that the saturating part of the vascular filling curve is not equivalent to the saturating part of the schematic FS curve, as commonly assumed. The former corresponds to a stabilized behavior while the latter is a rapid response. As explained above, the saturating part of the vascular filling curve occurs because the resistance to blood ejection increases highly, and even if the produced force and ventricular pressure also increase, a stroke volume saturation is observed. In other words, the LDA effect is overshadowed for high stressed blood volume values, but there is still an “LDA reserve”. Of course, our vascular filling simulations do not consider other regulatory mechanisms that would take place in reaction to fluid infusion, but they highlighted the importance of monitoring afterload and not only preload to predict fluid responsiveness. Also, these simulations were performed on an “healthy patient”, and do not consider a potentially altered LDA response. For instance, we may expect different fluid responsiveness curves from patients with heart failure or with dilated or hypertrophic cardiomyopathies.

Our CVS model presents several limitations that must be addressed. Regarding the cell model, we used a myofilament-based LDA that does not take into account the OFF state of myosin [29], nor the length-dependent changes in calcium transient [56]. However, this model still correctly reproduces the length-tension relationship (Fig 4) and could be used to link this cellular property to the organ and system scales. The ventricular model is rather simple, as it does not take into the complex, three-dimensional contraction of the ventricular chamber. However, force and pressure are still connected through geometrical laws (see S1 Appendix), and thus a geometrical effect is still accounted for in our model. In other words, there is still a physiological link between force and pressure in our model, but the ventricular response to preload changes would be better quantified with a more accurate model for the ventricular cavity. Also, it should be noted that the atria were not considered in the circulation model. However, as the ventricles hold the major role in ejecting the blood through the systemic and pulmonary circulations, adding atrial contraction in the circulation model should not qualitatively change our results. Despite these modeling simplifications, the model correctly reproduces baseline results and can help investigating multiscale questions. We performed a simple study to untangle the nature of the relationship between the FS mechanism (global behavior) and LDA (cellular property). We could then challenge the assumed link between the FS mechanism and vascular filling therapy. Of course, our model could be improved with a more accurate depiction of LDA at the sarcomere scale (by adding the myosin OFF state, for instance) and a better description of the ventricular geometry at the organ scale. However, we believe that a more accurate model would not challenge our qualitative results or question the multiscale nature of the studied phenomena. But of course, more precise models would provide better quantitative predictions about the relationship between LDA and the FS or fluid responsiveness mechanisms.

## Conclusion

**Fig 14:**
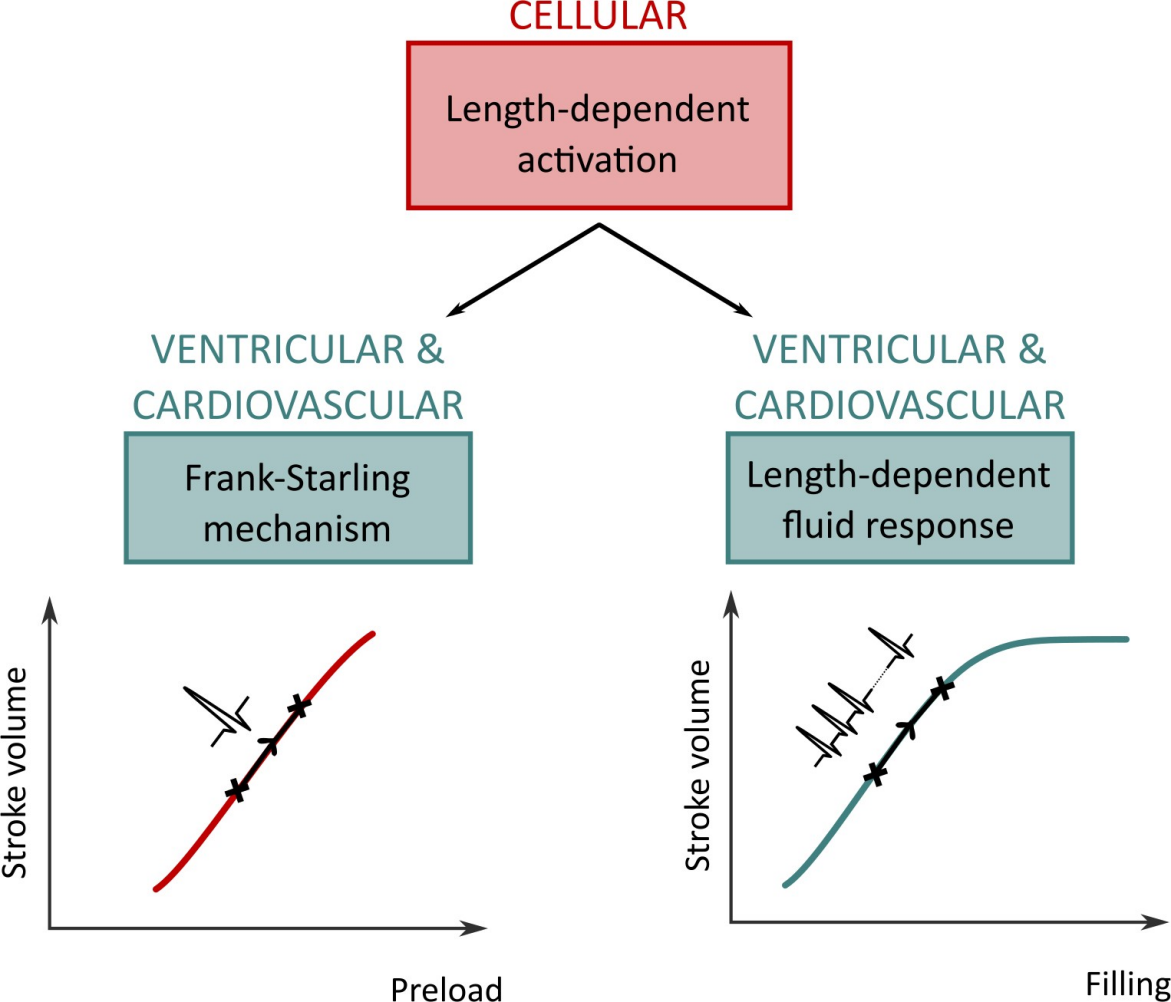
Visual representation of the interplay between the FS mechanism, vascular filling, and LDA. LDA, a cellular property, underlies the FS mechanism. The latter occurs at the organ scale on a beat-to-beat basis. LDA also underlies the motivation for vascular filling therapy. As vascular filling takes place, a new steady state is obtained after several heartbeats and a new stroke volume is achieved. Stroke volume, preload, and filling are variables which also impact or depend on cardiovascular-level variables, such as afterload. Each curve is a qualitative curve, as this figure is built from the qualitative conclusions of our study.

The heart is a complex multiphysics and multiscale system. Microscopic events at the subcellular level generate emergent properties at the organ scale [47,48]. For instance, the length-dependent activation, a property of cardiac cells, appears to underlie two important cardiac regulation mechanisms. The first one is the Frank-Starling mechanism, a rapid response to preload changes that occurs on a beat-to-beat basis. The second one is the length-dependent fluid response, a slower response to vascular filling. However, some misconceptions may occur in cardiology books or scientific literature and lead to a distorted understanding of the phenomena. In particular, the FS mechanism is sometimes presented as the underlying cause of vascular filling responses. It is true that both mechanisms share the same cellular origin, which is LDA. Furthermore, they both represent a complex, multiscale response to preload variations. But this is not enough to state that one mechanism causes the other. Our *in silico* multiscale study aimed to untangle those three concepts (FS mechanism, vascular filling therapy, and LDA) and provided a formal framework to characterize them. We emphasized that the three cellular, ventricular and cardiovascular scales participate truly in the global system behavior, and we showed that the roles of these three scales were different in the FS mechanism and in the vascular filling therapy. For instance, the aortic pressure at aortic valve opening changes as a function of preload for the vascular filling simulations, and not for the FS mechanism simulations. Thus, the positive response to vascular filling cannot always be linked to a positive response to an acute preload increase, as the complex interactions taking place are different in the two situations. A visual representation of our conclusions is shown in Fig 14. As our knowledge on cardiac function grows, we believe it is important to avoid misconceptions and keep a strict structure for defining cardiac concepts and properties. This contributes to a more comprehensive knowledge of the sophisticated length-dependent properties of cardiac muscle.

## Supporting information

S1 Appendix Model equations and parameters. **Table A.** Electrophysiological parameters. **Table B.** Mechanical parameters. **Table C.** Hemodynamic parameters. **Table D:** Standard values of hemodynamic quantities corresponding to a healthy subject that are used in the 13-parameter identification procedure. **Table E.** Standard values of hemodynamic quantities corresponding to a healthy subject that are used in the 9-parameter identification procedure.
(PDF)

S2 Appendix Analysis of the time evolution of the system during an IIP protocol. **Fig A.** Cellular variables during a heartbeat in the baseline case (solid blue), during an IIP protocol (solid red), and during an IIP protocol with NO LDA (dashed red). Vertical bars indicate intervals for the four phases of the cardiac cycle.
(PDF)

## Abbreviations

BL: Baseline
CB: Crossbridge
CVS: Cardiovascular System
FS: Frank-Starling
f: Crossbridges attachment rate
g_d_: Crossbridges irreversible detachment rate
IIP: Instantaneous Increase in Preload
IIP NO LDA: Instantaneous Increase in Preload when LDA is turned off
L: Half-sarcomere length
L_m_: Total half-sarcomere length (half-sarcomere length + series compliance length)
LDA: Length-dependent Activation
LDFR: Length-dependent fluid response
LV: Left ventricle
P_ao_: Aortic Pressure
P_lv_: Left Ventricular Pressure
PV: Pressure-Volume
Q_ao_: Aortic Flow
