# Supplementary material for: Frank-Starling mechanism, fluid responsiveness, and length-dependent activation: Unravelling the multiscale behaviors with an in silico analysis
Source: PLoS Comput Biol. 2021 Oct 11;17(10):e1009469. doi: 10.1371/journal.pcbi.1009469 (PMC8504729; doi:10.1371/journal.pcbi.1009469)
Supplement: S1 Appendix — Table A. Electrophysiological parameters. Table B. Mechanical parameters. Table C. Hemodynamic parameters. Table D: Standard values of hemodynamic quantities corresponding to a healthy subject that are used in the 13-parameter identification procedure. Table E. Standard values of hemodynamic quantities corresponding to a healthy subject that are used in the 9-parameter identification procedure. (PDF) [file pcbi.1009469.s001.pdf]

S1 - MODEL EQUATIONS

EQUATIONS GOVERNING THE CELLULAR ELECTROPHYSIOLOGICAL MODEL

Physical units used: time (t) in ms, membrane potential (V) in mV, transmembrane current densities (I_x) in pA/pF and intracellular and extracellular ionic concentrations (X_i and X_o) in µM (or µmol/L).

TRANSMEMBRANE CURRENTS AND MEMBRANE POTENTIAL [1, 2]

The time evolution of the membrane potential is described as follows:

$$\frac{dV}{dt} + I_{ion} + I_{stim} = 0$$

where I_{ion} is the sum of all transmembrane ionic current densities (see equation and figure below) and I_{stim} is the externally applied stimulus current density.

$$I_{ion} = I_{Na} + I_{K1} + I_{to} + I_{Kr} + I_{CaL} + I_{NaCa} + I_{NaK} + I_{pCa} + I_{pK} + I_{bCa} + I_{bNa}$$

$$I_{stim} = \begin{cases} -9.5 & \text{if } 100 < t < 106 \\ 0 & \text{otherwise} \end{cases}$$

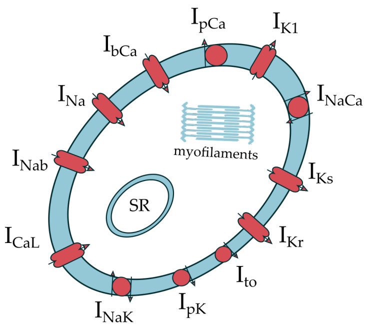

(a) Schematic representation of the transmembrane ionic currents described in the electrophysiological model.

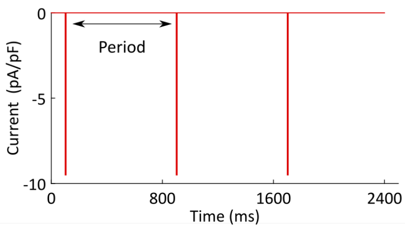

(b) Stimulus current used to trigger the AP. The stimulation period fixes the heart rate.

Nernst potentials

$$E_X = \frac{RT}{zF}\log\frac{[X]_o}{[X]_i} \quad \text{for } X = Na^+, Ca^{2+}, K^+$$

Fast sodium current: I_{Na}

$$m_\infty = \frac{1}{(1+\exp(\frac{-56.86-V}{9.03}))^2}$$

$$\alpha_m = \frac{1}{1+\exp(\frac{-60-V}{5})}$$

$$\beta_m = \frac{0.1}{1+\exp(\frac{V+35}{5})} + \frac{0.1}{1+\exp(\frac{V-50}{200})}$$

$$\tau_\infty = \alpha_m \beta_m$$

$$\frac{dm}{dt} = \frac{m_\infty - m}{\tau_\infty}$$

$$h_\infty = \frac{1}{(1+\exp(\frac{71.55+V}{7.43}))^2}$$

$$\alpha_h = \begin{cases} 0.057\exp(\frac{-80-V}{6.8}) & \text{if } V < 40 \\ 0 & \text{otherwise} \end{cases}$$

$$\beta_h = \begin{cases} 2.7\exp(0.079V) + 3.1\ 10^5\exp(0.3485V) & \text{if } V < 40 \\ \frac{0.77}{0.13(1+\exp(\frac{-V-10.66}{11.1}))} & \text{otherwise} \end{cases}$$

$$\tau_h = \frac{1}{\alpha_h + \beta_h}$$

$$\frac{dh}{dt} = \frac{h_\infty - h}{\tau_h}$$

$$j_\infty = \frac{1}{(1+\exp(\frac{71.55+V}{7.43}))^2}$$

$$\omega = -2.5428\ 10^4\exp(0.2444V) - 6.948\ 10^{-6}\exp(-0.04391V)$$

$$\alpha_j = \begin{cases} \frac{\omega(V+37.78)}{1+\exp(0.311(V+79.23))} & \text{if } V < 40 \\ 0 & \text{otherwise} \end{cases}$$

$$\beta_j = \begin{cases} \frac{0.02424\exp(-0.01052V)}{1+\exp(-0.1378(V+40.14))} & \text{if V} < 40 \\ \frac{0.6\exp(0.057V)}{1+\exp(-0.1(V+32))} & \text{otherwise} \end{cases}$$

$$\tau_j = \frac{1}{\alpha_j + \beta_j}$$

$$\frac{dj}{dt} = \frac{j_\infty - j}{\tau_j}$$

$$I_{Na} = G_{Na} m^3 hj(V - E_{Na})$$

L-type calcium current: I_{CaL}

$$d_\infty = \frac{1}{1+\exp(\frac{-8-V}{7.5})}$$

$$\alpha_d = \frac{1.4}{1+\exp(\frac{-35-V}{13})} + 0.25$$

$$\beta_d = \frac{1.4}{1+\exp(\frac{5+V}{5})}$$

$$\gamma_d = \frac{1}{1+\exp(\frac{50-V}{20})}$$

$$\tau_d = \alpha_d \beta_d + \gamma_d$$

$$\frac{dd}{dt} = \frac{d_\infty - j}{\tau_d}$$

$$f_\infty = \frac{1}{1+\exp(\frac{20+V}{7})}$$

$$\alpha_f = 1102.5\exp(\frac{-(27+V)^2}{225})$$

$$\beta_f = \frac{200}{1+\exp(\frac{13-V}{10})}$$

$$\gamma_f = \frac{180}{1+\exp(\frac{30+V}{10})} + 20$$

$$\tau_f = \alpha_f + \beta_f + \gamma_f$$

$$\frac{df}{dt} = \frac{f_\infty - f}{\tau_f}$$

$$f_{2\infty} = 0.33 + \frac{0.67}{1+\exp(\frac{35+V}{7})}$$

$$\alpha_{f_2} = 562\exp(-\frac{(V+27)^2}{240})$$

$$\beta_{f_2} = \frac{31}{1+\exp(\frac{25-V}{10})}$$

$$\gamma_{f_2} = \frac{80}{1+\exp(\frac{30+V}{10})}$$

$$\tau_{f_2} = \alpha_{f_2} + \beta_{f_2} + \gamma_{f_2}$$

$$\frac{df_2}{dt} = \frac{f_{2\infty} - f_2}{\tau_{f_2}}$$

$$f_{cass\infty} = \frac{0.6}{1+(\frac{Ca_{ss}}{50})^2} + 0.4$$

$$\tau_{fcass} = 2 + \frac{80}{1+(\frac{Ca_{ss}}{50})^2}$$

$$\frac{df_{Ca_{ss}}}{dt} = \frac{f_{cass\infty} - f_{Ca_{ss}}}{\tau_{fcass}}$$

$$I_{CaL} = G_{CaL} d\, f\, f_2\, f_{Ca_{ss}}\, 4 \frac{(V-15)F^2}{RT} \times \frac{0.25\, Ca_{ss} \exp(\frac{2(V-15)F}{RT}) - Ca_o}{1000(\exp(\frac{2(V-15)F}{RT}) - 1)}$$

Transient outward potassium current: I_{to}

$$r_\infty = \frac{1}{1+\exp(\frac{20-V}{6})}$$

$$\tau_r = 9.5 \exp(-\frac{(V+40)^2}{1800}) + 0.8$$

$$\frac{dr}{dt} = \frac{r_\infty - r}{\tau_r}$$

$$s_\infty = \frac{1}{1+\exp(\frac{20+V}{5})}$$

$$\tau_s = 85 \exp(-\frac{(V+45)^2}{320}) + \frac{5}{1+\exp(\frac{V-20}{5})} + 3$$

$$\frac{ds}{dt} = \frac{s_\infty - r}{\tau_s}$$

$$I_{to} = G_{to} r s (V - E_K)$$

Slow delayed rectifier current: I_{Ks}

$$x_{s\infty} = \frac{1}{1+\exp(\frac{-5-V}{14})}$$

$$\alpha_{xs} = \frac{1400}{\sqrt{1+\exp(\frac{-5-V}{6})}}$$

$$\beta_{xs} = \frac{1}{1+\exp(\frac{V-35}{15})}$$

$$\tau_{xs} = \alpha_{xs}\beta_{xs} + 80$$

$$E_{Ks} = \frac{RT}{F} \log(\frac{K_o + p_{KNa} Na_o}{K_i + p_{KNa} Na_i})$$

$$I_{Ks} = G_{Ks} x_s^2 (V - E_{Ks})$$

Rapid delayed rectifier current: I_{Kr}

$$x_{r1\infty} = \frac{1}{1+\exp(\frac{-26-V}{7})}$$

$$\alpha_{r1} = \frac{450}{1+\exp(\frac{-45-V}{10})}$$

$$\beta_{r1} = \frac{6}{1+\exp(\frac{30+V}{11.5})}$$

$$\tau_{r1} = \alpha_{r1}\beta_{r1}$$

$$\frac{dx_{r1}}{dt} = \frac{x_{r1\infty} - x_{r1}}{\tau_{r1}}$$

$$x_{r2\infty} = \frac{1}{1+\exp(\frac{V+88}{24})}$$

$$\alpha_{r2} = \frac{3}{1+\exp(\frac{-60-V}{20})}$$

$$\beta_{r2} = \frac{1.12}{1+\exp(\frac{-60+V}{20})}$$

$$\tau_{r2} = \alpha_{r2}\beta_{r2}$$

$$\frac{dx_{r1}}{dt} = \frac{x_{r1\infty} - x_{r1}}{\tau_{r1}}$$

$$I_{Kr} = G_{Kr}\sqrt{\frac{K_o}{5400}}x_{r1}x_{r2}(V - E_K)$$

Inward rectifier potassium current: I_{K1}

$$\alpha_{K1} = \frac{0.1}{1+\exp(0.06(V - E_K - 200))}$$

$$\beta_{K1} = \frac{3\exp(0.0002(V - E_K + 100)) + \exp(0.1(V - E_K - 10))}{1+\exp(-0.5(V - E_K))}$$

$$x_{K1\infty} = \frac{\alpha_{K1}}{\alpha_{K1} + \beta_{K1}}$$

$$I_{K1} = G_{K1}x_{K1\infty}(V - E_K)$$

Na/Ca exchanger current: I_{NaCa}

$$I_{NaCa} = \frac{k_{NaCa}}{K_{mN}^3 + Na_o^3} \times \frac{\exp(\frac{\gamma VF}{RT})Na_i^3Ca_o - 2.5\exp(\frac{(1-\gamma)VF}{RT})Na_o^3Ca_i}{(K_{mCa} + Ca_o)(1 + k_{sat}\exp(\frac{(\gamma-1)VF}{RT}))}$$

Na/K pump current: I_{NaK}

$$I_{NaK} = \frac{P_{NaK}K_oNa_i}{(K_o + K_{mK})(Na_i + K_{mNa})} \times \frac{1}{(1 + 0.1245\exp(\frac{-0.1VF}{RT}) + 0.0353\exp(\frac{-VF}{RT}))}$$

Sarcolemmal calcium pump current: I_{pCa}

$$I_{pCa} = G_{pCa} \frac{Ca_i}{K_{pCa} + Ca_i}$$

Sarcolemmal potassium pump current: I_{pK}

$$I_{pK} = G_{pK} \frac{V - E_K}{1 + \exp(\frac{25 - V}{5.98})}$$

Background currents: I_{bNa} *and* I_{bCa}

$$I_{bNa} = G_{bNa}(V - E_{Na})$$
$$I_{bCa} = G_{bCa}(V - E_{Ca})$$

CALCIUM DYNAMICS

Calcium dynamics is described within three subcellular compartments (see figure): cytoplasm (CYTO), sarcoplasmic reticulum (SR) and diadic subspace (SS). In the following equations, $[Ca]_i$ is free cytoplasmic calcium concentration, $[Ca]_{SR}$ is free SR calcium concentration and $[Ca]_{ss}$ is free diadic subspace calcium concentration (all concentrations expressed in μM). I_{up}, I_{rel}, I_{xfer} and I_{leak} are expressed in μM/ms.

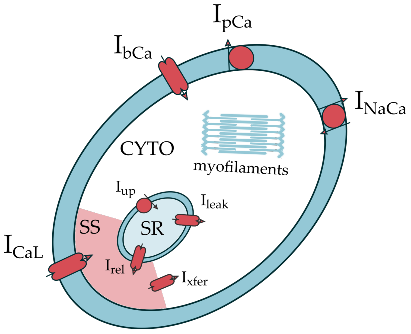

- SERCA (ten Tusscher and Panfilov [2])

$$I_{up} = \frac{V_{up}}{1 + (\frac{K_{up}}{Ca_i})^2}$$

- CICR (Lascano *et al.* [3])

$$u = max_{sr} - \frac{max_{sr} - min_{sr}}{1 + (\frac{EC_{50}}{Ca_{SR}})^2}$$

$$k_1 = \frac{k_1'}{u}, \; k_2 = k_2' u$$

$$
\begin{aligned}
RI &= 1 - R - O - I \\
\frac{dR}{dt} &= k_4 RI - k_2 RCa_{ss} \\
&- k_1 RCa_i{}^2 + k_3 O \\
\frac{dO}{dt} &= k_1 RCa_{ss}{}^2 \\
&- k_3 O - k_2 OCa_{ss} + k_4 I \\
\frac{dI}{dt} &= k_2 OCa_{ss} - k_4 I \\
&- k_3 I + k_1 RICa_{ss}^2
\end{aligned}
$$

$$I_{rel} = V_{rel} O (Ca_{SR} - Ca_{ss}) + V_{leak(d)} R (Ca_{SR} - Ca_{ss})$$

$$I_{leak} = V_{leak(c)} (Ca_{SR} - Ca_i)$$

$$I_{xfer} = V_{xfer} (Ca_{ss} - Ca_i)$$

- Mechanical feedback (Kosta *et al.* [4])

$$
\begin{aligned}
I_{trop} &= \frac{1}{2}\, 3\, \left(\frac{d[TSCa_3]_{LV}}{dt} + \frac{d[TSCa_3^{\sim}]_{LV}}{dt} + \frac{d[TSCa_3^{*}]_{LV}}{dt}\right) \\
&+ \frac{1}{2}\, 3\, \left(\frac{d[TSCa_3]_{RV}}{dt} + \frac{d[TSCa_3^{\sim}]_{RV}}{dt} + \frac{d[TSCa_3^{*}]_{RV}}{dt}\right)
\end{aligned}
$$

  The colored concentrations are obtained from the sarcomere contraction model.

- Free calcium concentrations in the CYTO, SR and SS compartments

$$
\begin{aligned}
\frac{d[Ca]_i}{dt} &= -\frac{1}{1 + \dfrac{K_{buf_i}\, Buf_i}{([Ca]_i + K_{buf_i})^2}} \left(C_{m0} \frac{I_{bCa} + I_{pCa} - 2 I_{NaCa}}{2 V_c F} \right. \\
&\left. + \frac{V_{sr}}{V_c} (I_{leak} - I_{up}) + I_{xfer} - I_{trop} \right)
\end{aligned}
$$

$$\frac{d[Ca]_{SR}}{dt} = -\frac{1}{1 + \dfrac{K_{bufSR}\, Buf_{SR}}{([Ca]_{SR} + K_{bufSR})^2}} (I_{up} - I_{leak} - I_{rel})$$

$$\frac{d[Ca]_{SS}}{dt} = -\frac{1}{1 + \dfrac{K_{bufSS}\, Buf_{SS}}{([Ca]_{SS} + K_{bufSS})^2}} \left(-C_{m0} \frac{I_{CaL}}{2 V_{ss} F} + \frac{V_{sr}}{V_{ss}} I_{rel} - \frac{V_c}{V_{ss}} I_{xfer} \right)$$

Sodium and potassium intracellular concentrations

$$
\begin{aligned}
\frac{d[K]_i}{dt} &= -C_{m0} \frac{-2 I_{NaK} + I_{Ks} + I_{K1} + I_{to} + I_{pK} - I_{stim}}{V_c F} \\
\frac{d[Na]_i}{dt} &= -C_{m0} \frac{3 I_{NaK} + 3 I_{NaCa} + I_{Na} + I_{bNa}}{V_c F}
\end{aligned}
$$

Parameter	Units	Value	Parameter	Units	Value
R	J/K/mol	8.314472	K_{mNa}	μM	40 000
F	C/mol	96485.3415	G_{pCa}	nS/pF	0.1238
T	K	310	K_{pCa}	μM	0.5
C_{m0}	μF	1.84 10^{-4}	G_{pK}	nS/pF	0.0146
V_c	mL	1.6404 10^{-8}	V_{up}	μM/ms	6.375
V_{SR}	mL	1.0941 10^{-9}	K_{up}	μM	0.25
V_{SS}	mL	5.4681 10^{-11}	EC_{50}	μM	1500
K_o	μM	5400	k_1'	$μM^2$/ms	1.5
Na_o	μM	140 000	k_2'	μM/ms	4.5
Ca_o	μM	2000	k_3	ms^{-1}	0.06
G_{Na}	nS/pF	14.838	k_4	ms^{-1}	0.005
G_{CaL}	cm^3/ms/μF	3.98 10^{-5}	V_{rel}	ms^{-1}	0.102
G_{to}	nS/pF	0.294	$V_{leak(d)}$	ms^{-1}	1.8 10^{-5}
G_{Ks}	nS/pF	0.098	$V_{leak(c)}$	ms^{-1}	3.42 10^{-4}
p_{KNa}	-	0.03	V_{xfer}	ms^{-1}	0.0038
G_{Kr}	nS/pF	0.153	Buf_i	μM	130
G_{K1}	nS/pF	5.405	K_{buf_i}	μM	1
k_{NaCa}	pA/pF	1000	Buf_{SR}	μM	10^4
K_{mN}	μM	87 500	K_{bufSR}	μM	300
γ	-	0.35	Buf_{SS}	μM	400
k_{sat}	-	0.1	K_{bufSS}	μM	0.25
P_{NaK}	pA/pF	2.724	Period	ms	800
K_{mK}	μM	1000			

Table A: Electrophysiological parameters

EQUATIONS GOVERNING THE SARCOMERE CONTRACTION MODEL

LEFT VENTRICLE

The "LV" indices below designate the left ventricle.

Calcium kinetics

$$[TS]_{LV} = [TS]_{t,LV} - [TSCa_3]_{LV} - [TSCa_3^{\sim}]_{LV} - [TSCa_3^{*}]_{LV} - [TS^{*}]_{LV}$$

$$\begin{aligned}
\frac{d[TSCa_3]_{LV}}{dt} &= Y_b[TS]_{LV}[Ca]_i{}^3 - Z_b[TSCa_3]_{LV} + g[TSCa_3^{\sim}]_{LV} - f[TSCa_3]_{LV} \\
\frac{d[TSCa_3^{\sim}]_{LV}}{dt} &= f[TSCa_3]_{LV} - g[TSCa_3^{\sim}]_{LV} + Z_p[TSCa_3^{*}]_{LV} - Y_p[TSCa_3^{\sim}]_{LV} \\
\frac{d[TSCa_3^{*}]_{LV}}{dt} &= Y_p[TSCa_3^{\sim}]_{LV} - Z_p[TSCa_3^{*}]_{LV} + Z_r[TS^{*}]_{LV}[Ca]_i{}^3 - Y_r[TSCa_3^{*}]_{LV} \\
\frac{d[TS^{*}]_{LV}}{dt} &= Y_r[TSCa_3^{*}]_{LV} - Z_r[TS^{*}]_{LV}[Ca]_i{}^3 - g_d[TS^{*}]_{LV}
\end{aligned}$$

Intracellular calcium concentration ($[Ca]_i$) is obtained from the electrophysiological model.

$$f = Y_a \exp(-R_{La}(L_{LV} - L_a)^2)$$

$$g = Z_a + Y_h$$

$$Y_h = \begin{cases} Y_v(1 - \exp(-\gamma(h_{w_{LV}} - h_{wr_{LV}})^2)) & \text{if } h_{w_{LV}} < h_{wr_{LV}} \\ 0.1Y_v(1 - \exp(-\gamma(h_{w_{LV}} - h_{wr_{LV}})^2)) & \text{otherwise} \end{cases}$$

$$g_d = Y_d \exp(-Y_c(L_{LV} - L_c))$$

Half-sarcomere length and force

$$h_{w_{LV}} = L_{LV} - X_{w_{LV}}$$
$$h_{p_{LV}} = L_{LV} - X_{p_{LV}}$$

$$\frac{dX_{w_{LV}}}{dt} = B_w(h_{w_{LV}} - h_{wr_{LV}})$$
$$\frac{dX_{p_{LV}}}{dt} = B_p(h_{p_{LV}} - h_{pr_{LV}})$$

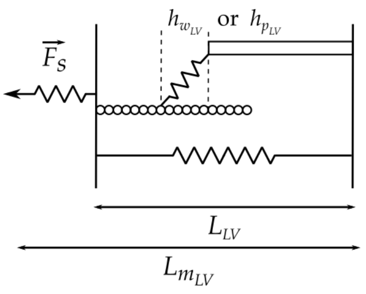

$$
\begin{aligned}
F_a &= A_w\,[\mathrm{TSCa}_3^{\sim}]_{\mathrm{LV}}\,h_{w_{LV}} + A_p\,([\mathrm{TSCa}_3^{*}]_{\mathrm{LV}} + [\mathrm{TS}^{*}]_{\mathrm{LV}})\,h_{p_{LV}}\\
F_p &= K_e(L_{LV} - L_0)^5 + L_e(L_{LV} - L_0)\\
F &= F_a + F_p\\
F_s &= \alpha(\exp(\beta(L_{m,LV} - L_{LV})) - 1)
\end{aligned}
$$

$L_{m,LV}$ is obtained from the hemodynamic model and L_{LV} is calculated for each iteration step by solving $F - F_s = 0$, using the MatLab *fzero* function.

RIGHT VENTRICLE (RV)

The set of equations for RV sarcometric contraction is the same as for LV, where every "LV" subscript has to be replaced with "RV". The parameters listed below are identical for both ventricles.

Parameter	Units	Value
A_p	$mN/mm^2/\mu m/\mu M$	2850
A_w	$mN/mm^2/\mu m/\mu M$	570
α	mN/mm^2	0.15
β	ms^{-1}	80
B_p	ms^{-1}	1.75
B_p	ms^{-1}	1.225
Y_a	ms^{-1}	$2.3\,10^{-3}$
Y_b	$\mu M^{-3}.ms^{-1}$	0.1816
Y_c	μM^{-1}	4
Y_d	ms^{-1}	0.028
Y_p	ms^{-1}	0.1397
Y_r	ms^{-1}	0.1397
Y_v	ms^{-1}	0.9
Z_a	ms^{-1}	0.0023
Z_b	ms^{-1}	0.1397
Z_p	ms^{-1}	0.2095
Z_r	$\mu M^{-3}.ms^{-1}$	7.2626
$[TS]_t$	μM	70/3
L_0	μm	0.97
L_a	μm	1.15
R	μm^{-2}	15
L_c	μm	1.05
h_{pr}	μm	$6\ 10^{-3}$
h_{wr}	μm	10^{-4}
γ	ms^{-1}	$2.8\ 10^{-4}$
K_e	$mN/mm^2/\mu m^{-5}$	$3.15\ 10^4$
L_e	$mN/mm^2/\mu m$	3

Table B: Mechanical parameters

EQUATIONS GOVERNING THE HEMODYNAMIC (6-CHAMBER) MODEL

PASSIVE CHAMBERS PRESSURES

$$P_{ao} = E_{ao} \cdot V_{ao} \quad \text{with } E_{ao} = 1/C_{ao}$$
$$P_{vc} = E_{vc} \cdot V_{vc} \quad \text{with } E_{vc} = 1/C_{vc}$$
$$P_{pa} = E_{pa} \cdot V_{pa} \quad \text{with } E_{pa} = 1/C_{pa}$$
$$P_{pv} = E_{pv} \cdot V_{pv} \quad \text{with } E_{pv} = 1/C_{pv}$$

FLOWS

- Pulmonary and systemic flows:

$$Q_{sys} = \frac{P_{ao} - P_{vc}}{R_{sys}}$$
$$Q_{pul} = \frac{P_{pa} - P_{pv}}{R_{pul}}$$

- Valves flows:

$$Q_{mt} = \begin{cases} \dfrac{P_{pv} - P_{lv}}{R_{mt}} & \text{if } P_{PV} > P_{LV} \\ 0 & \text{otherwise} \end{cases}$$

$$Q_{av} = \begin{cases} \dfrac{P_{lv} - P_{ao}}{R_{av}} & \text{if } P_{LV} > P_{AO} \\ 0 & \text{otherwise} \end{cases}$$

$$Q_{tc} = \begin{cases} \dfrac{P_{vc} - P_{rv}}{R_{tc}} & \text{if } P_{VC} > P_{RV} \\ 0 & \text{otherwise} \end{cases}$$

$$Q_{pv} = \begin{cases} \dfrac{P_{rv} - P_{pa}}{R_{pv}} & \text{if } P_{RV} > P_{PA} \\ 0 & \text{otherwise} \end{cases}$$

VOLUMES

$$\frac{dV_{lv}}{dt} = Q_{mt} - Q_{av}$$
$$\frac{dV_{ao}}{dt} = Q_{av} - Q_{sys}$$
$$\frac{dV_{vc}}{dt} = Q_{sys} - Q_{tc}$$
$$\frac{dV_{rv}}{dt} = Q_{tc} - Q_{pv}$$
$$\frac{dV_{pa}}{dt} = Q_{pv} - Q_{pul}$$

$$V_{pv} = SBV - V_{lv} - V_{ao} - V_{vc} - V_{rv} - V_{pa}$$

ACTIVE CHAMBERS PRESSURES

LV (RV) ventricle is assimilated to a thick walled sphere (see figure below) with a wall volume $V_{w_{LV}}$ ($V_{w_{RV}}$). The reference (and variable) radius R_{LV} (R_{RV}) corresponds to the position of the N_{LV} (N_{RV}) sarcomeres of length $L_{m,LV}$ ($L_{m,RV}$) assembled in circle inside the ventricular wall. The volume included between $r_{in_{LV}}$ and R_{LV} ($r_{in_{RV}}$ and R_{RV}) is noted $V_{r_{LV}}$ ($V_{r_{RV}}$).

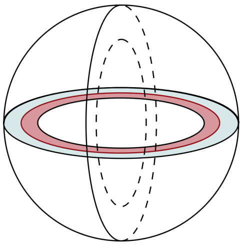

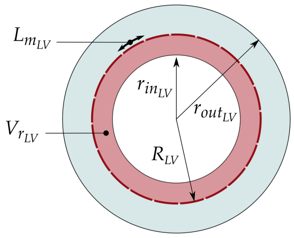

$$\frac{4}{3}\pi r_{in_{LV}}^3 = V_{LV} \Rightarrow r_{in_{LV}} = \sqrt[3]{\frac{3}{4\pi}V_{LV}}$$
$$\frac{4}{3}\pi r_{out_{LV}}^3 = V_{LV} + V_{w_{LV}} \Rightarrow r_{out_{LV}} = \sqrt[3]{\frac{3}{4\pi}V_{LV} + V_{w_{LV}}}$$
$$\frac{4}{3}\pi R_{LV}^3 = V_{LV} + V_{r_{LV}} \Rightarrow R_{LV} = \sqrt[3]{\frac{3}{4\pi}V_{LV} + V_{r_{LV}}}$$
$$N_{LV}\, L_{m,LV} = 2\,\pi\, R_{LV} \Rightarrow L_{m,LV} = \frac{2\pi R_{LV}}{N_{LV}}$$

$$P_{LV} = 7.5\, F_{LV}\, \frac{L_{m,LV}}{L_r} \left((\frac{r_{out_{LV}}}{r_{in_{LV}}})^2 - 1 \right) + \lambda\, (V_{LV} - V_0)$$

Parameter	Units	Value
SBV	ml	940.86 (identified*)
R_{sys}	10^3 mmHg · ms/ml	1.38 (identified)
R_{pul}	mmHg · ms/ml	109.57 (identified*)
R_{mt}	mmHg · ms/ml	22.09 (identified)
R_{tc}	mmHg · ms/ml	11.56 (identified)
R_{av}	mmHg · ms/ml	47.96 (identified)
R_{pv}	mmHg · ms/ml	3.51 (identified)
C_{ao}	mmHg/ml	0.9550 (identified*)
C_{vc}	mmHg/ml	79.10 (identified)
C_{pa}	mmHg/ml	2.43 (identified*)
C_{pv}	mmHg/ml	23.34 (identified)
V_{lvw}	ml	334.86 (identified*)
V_{rvw}	ml	48.31 (identified*)
λ	mmHg/ml^3	5 10^{-5} [5]
V_0	ml	80 [5]

Table C: Hemodynamic parameters.
* The identified parameter has a different value than reported in [4], because a two-step identification procedure was used (see explanations in text).

F_{LV} is obtained from the sarcomere contraction model.
The same reasoning for the RV gives:

$$P_{RV} = 7.5\, F_{RV}\, \frac{Lm_{RV}}{L_r} \left((\frac{r_{out_{RV}}}{r_{in_{RV}}})^2 - 1 \right) + \lambda\, (V_{RV} - V_0)$$

Parameters optimization

Thirteen hemodynamic parameters from the cardiovascular system have to be identified, and they are listed in Table C: the four valves resistances, the two circulation resistances, the four passive chambers compliances, wall volume of both ventricles, and stressed blood volume. The parameters are identified using the *fminsearch* algorithm from MATLAB (The MathWorks, Natick, MA, USA) in order to minimize an objective function defined as the absolute relative error between a chosen set of reference variables and their corresponding calculated values. The value for the reference data were chosen in order to correspond to standard healthy values [6–8] and are given in Table D and E.

Our optimization procedure consists in the following two-step approach. First note that it is well known that valve resistances are

practically difficult to identify, but their precise values do not a play an important role in the final model. For this reason, they are generally excluded from the identification procedure of hemodynamic models [9–11]. To obtain an estimate for the valve resistances, we perform a first identification procedure for the 13 parameters listed in Table C by introducing the data from Table D in our objective function. Then we fix the obtained valve resistance values, and we perform a second identification procedure with the remaining nine parameters and the reference data from Table E.

	Standard value	Units
Stroke volume	60	ml
Maximal LV pressure	120	mmHg
Maximal RV pressure	21,5	mmHg
Maximal aortic pressure	113	mmHg
Amplitude of aortic pressure	35	mmHg
Maximal pulmonary artery pressure	21	mmHg
Amplitude of pulmonary artery pressure	13	mmHg
Maximal pulmonary vein pressure	7,5	mmHg
Amplitude of pulmonary vein pressure	2	mmHg
Maximal vena cava pressure	7	mmHg
Amplitude of vena cava pressure	0,5	mmHg
Minimal LV volume	60	ml
Minimal RV volume	60	ml

Table D: Standard values of hemodynamic quantities corresponding to a healthy subject that are used in the 13-parameter identification procedure (see text).

	Standard value	Units
Stroke volume	60	ml
Mean LV pressure	59	mmHg
Mean RV pressure	12	mmHg
Mean aortic pressure	108	mmHg
Mean pulmonary artery pressure	19	mmHg
Amplitude of pulmonary vein pressure	2	mmHg
Amplitude of vena cava pressure	0.5	mmHg
Minimal LV volume	60	ml
Minimal RV volume	60	ml

Table E: Standard values of hemodynamic quantities corresponding to a healthy subject that are used in the 9-parameter identification procedure (see text).

[1] K. H. W. J. ten Tusscher, D. Noble, P. J. Noble, and A. V. Panfilov, "A model for human ventricular tissue," *Am. J. Physiol. Circ. Physiol.*, vol. 286, no. 4, H1573–H1589, 2004, ISSN: 0363-6135.

[2] K. H. W. J. ten Tusscher and A. V. Panfilov, "Alternans and spiral breakup in a human ventricular tissue model.," *Am. J. Physiol. Heart Circ. Physiol.*, vol. 291, no. 3, H1088–100, 2006, ISSN: 0363-6135.

[3] E. C. Lascano, M. Said, L. Vittone, A. Mattiazzi, C. Mundiña-Weilenmann, and J. A. Negroni, "Role of CaMKII in post acidosis arrhythmias: a simulation study using a human myocyte model.," *J. Mol. Cell. Cardiol.*, vol. 60, pp. 172–83, 2013, ISSN: 1095-8584.

[4] S. Kosta, J. Negroni, E. Lascano, and P. Dauby, "Multiscale model of the human cardiovascular system: Description of heart failure and comparison of contractility indices," *Math. Biosci.*, vol. 284, pp. 71–79, 2017, ISSN: 0025-5564.

[5] J. A. Negroni and E. C. Lascano, "Concentration and Elongation of Attached Cross-bridges as Pressure Determinants in a Ventricular Model," *J. Mol. Cell. Cardiol.*, vol. 31, no. 8, pp. 1509–1526, 1999, ISSN: 0022-2828.

[6] *Edwards Lifesciences: Normal Hemodynamic Parameters and Laboratory Values*. [Online]. Available: https://edwardsprod.blob.core.windows.net/media/Gb/devices/monitoring/hemodynamic%20monitoring/normal_hemodynamic_parameters_pocket_card.pdf.

[7] *LiDCO - Normal Hemodynamic Parameters*. [Online]. Available: http://www.lidco.com/clinical/hemodynamic.php.

[8] R. E. Klabunde, *Cardiovascular physiology concepts*. Lippincott Williams & Wilkins, 2005, p. 235, ISBN: 078175030X.

[9] A. Pironet, "Model-Based Prediction of the Response to Vascular Filling Therapy," Ph.D. dissertation, Université de Liège, Liège, Belgique, 2016.

[10] A. Pironet, P. C. Dauby, J. G. Chase, P. D. Docherty, J. A. Revie, and T. Desaive, "Structural identifiability analysis of a cardiovascular system model," *Med. Eng. Phys.*, vol. 38, no. 5, pp. 433–441, 2016, ISSN: 13504533.

[11] J. A. Revie, D. J. Stevenson, J. G. Chase, C. E. Hann, B. C. Lambermont, A. Ghuysen, P. Kolh, G. M. Shaw, S. Heldmann, and T. Desaive, "Validation of subject-specific cardiovascular system models from porcine measurements," *Comput. Methods Programs Biomed.*, vol. 109, no. 2, pp. 197–210, 2013, ISSN: 01692607.
