# Supplementary material for: Frank-Starling mechanism, fluid responsiveness, and length-dependent activation: Unravelling the multiscale behaviors with an in silico analysis
Source: PLoS Comput Biol. 2021 Oct 11;17(10):e1009469. doi: 10.1371/journal.pcbi.1009469 (PMC8504729; doi:10.1371/journal.pcbi.1009469)
Supplement: S2 Appendix — Fig A. Cellular variables during a heartbeat in the baseline case (solid blue), during an IIP protocol (solid red), and during an IIP protocol with NO LDA (dashed red). Vertical bars indicate intervals for the four phases of the cardiac cycle. (PDF) [file pcbi.1009469.s002.pdf]

S2 - Analysis of the time evolution of the system during an IIP protocol

In the present section, the evolution of the cellular and ventricular variables during an instantaneous increase in preload (IIP) protocol is discussed. A comparison between the baseline (BL), IIP and IIP NO LDA cases is provided. One important aim of this discussion is to investigate why the active force increases upon IIP but does not lead to an increase in pressure in the IIP NO LDA case, as opposed to the IIP with LDA considered.

Crossbridges mechanics

The mean crossbridges elongations h_w and h_p depend on the half-sarcomere length L. Any variation in the half-sarcomere length L leads to instantaneous variations in h_w and h_p because they are linked as follows:

$$L = X_w + h_w = X_p + h_p \tag{1}$$

where X_w and X_p correspond to inextensible lengths.

Any time-variation of L impact the variation of h_w and h_p (= velocity dependence) according to the following equations:

$$\frac{dL}{dt} = \frac{dh_w}{dt} + B_w(h_w - h_{wr}) \tag{2}$$

$$\frac{dL}{dt} = \frac{dh_p}{dt} + B_p(h_p - h_{pr}) \tag{3}$$

where B_p, B_w, h_{wr} and h_{pr} are constant parameters.

Those length dependencies cannot be frozen in the NO LDA model, as they are part of the working hypotheses of the half-sarcomere model from [1].

Panels B, C and D from Figure A show how variations in L impact variations in h_w and h_p. The red curves (solid and dashed) correspond to an IIP protocol (with and without LDA respectively) with an increased L during the filling phase compared to the BL case (blue curves). h_w and h_p evolutions are thus different for the BL and both IIP cases.

Crossbridges concentrations

Crossbridges concentrations depend on the crossbridge cycle transition rates. The attachment rate f and irreversible detachment rate g_d depend on L (Eqs. 4-5), while the detachment rate g depend on the mean crossbridge elongation h_w (Eq. 6).

$$f = Y_a \exp(-R(L - L_a)^2) \tag{4}$$

$$g_d = Y_d \exp(-Y_c(L - L_c)) \tag{5}$$

$$g = Z_a + Y_h \tag{6}$$

where $Y_h = \begin{cases} Y_v(1 - \exp(-\gamma(h_w - h_{wr})^2)) & \text{if } \mathrm{h_w} < \mathrm{h_{wr}} \\ 0.1Y_v(1 - \exp(-\gamma(h_w - h_{wr})^2)) & \text{otherwise} \end{cases}$

The reversible detachment rate g depends on h_w, and thus indirectly depends on L time-variations (= velocity dependence, see Eq. 2).

Note that, in addition to the questions examined in the paper, we have also considered a "FIXED BIOCHEMISTRY" model where both the length and velocity dependencies were frozen. We observed that the stroke volume following an instantaneous increase in preload was decreased by 21,87% compared to the baseline case (data not shown). This result suggests that removing the length and velocity dependencies of the CB biochemical cycle hinders the positive adaptation of stroke volume to preload increase.

Panels E, F and G show how those transition rates vary during a heartbeat. The BL and IIP NO LDA cases have the same attachment and detachment rates, as the length-dependence for the NO LDA case was "frozen". The IIP LDA case shows a global increased attachment rate (panel E) and decreased irreversible detachment rate (panel G) compared to the BL case. The IIP NO LDA case presents a decreased detachment rate compared to the BL and IIP LDA cases (panel F), as h_w (panel C) is slightly higher compared to the two other curves. This leads to different attached crossbridges concentration evolutions (panel H) for the three cases, with the highest concentrations observed for the IIP LDA case and the lowest concentrations observed for the BL case.

In the filling and isovolumic contraction phases, as the calcium is still low (panel A) and activation occurs late in the isovolumic phase, the differences between the BL, IIP and IIP NO LDA behaviors are mainly "mechanical", that is, mainly

noticeable in the L, h_w and h_p evolutions (panels B, C and D), while crossbridges concentrations stay very similar (panel H). During the ejection phase, as L varies to a greater extent, and as crossbridges cycle through the different states, differences in the mechanical and biochemical variables become more marked between the three cases.

Force and pressure developments

Total force (panel I) is given by the sum of active and passive forces. The active force is given by the product of crossbridges elongations and crossbridges concentrations:

$$F = A_w\,[\mathrm{TSCa}_3^{\sim}]\,h_w + A_p\,([\mathrm{TSCa}_3^{*}] + [\mathrm{TS}^{*}])\,h_p \tag{7}$$

The crossbridges elongations are similar for the three cases (panels C and D), while the attached crossbridges concentrations are the highest for the IIP LDA case and the lowest for the BL case. It is thus expected to observe the lowest force values for the BL case, then IIP NO LDA, and finally the greatest force values for IIP LDA, as shown in panel I.

Panel J shows the time evolution of the total half-sarcomere length L_m. IIP NO LDA shows the lowest variations in L_m. This means that the IIP NO LDA case is responsible for the lowest ejected blood volume. This is due to the complex interactions between cellular variables (length and force) and ventricular variables (volume and pressure) subjected to hemodynamical constraints (preload and afterload). Those interdependent variables are altered when there is an increase in preload (see the differences in the BL and IIP LDA variables evolutions), but also when the length-dependence of biochemical rates are frozen (see the differences in the IIP LDA and IIP NO LDA cases).

Finally, ventricular pressure (panel K) depends on both the total produced force and L_m:

$$P_{lv} = 7,5\,F_m\,\frac{L_m}{L_r}\,\left((\frac{r_{out}}{r_{in}})^2 - 1\right) + \lambda\,(V_{lv} - V_0) \tag{8}$$

where r_{out} and r_{in} are the outer and inner radius of the spherical ventricle, which are linked to L_m.

An increased in force for the IIP NO LDA case compared to the BL case is "counter-balanced" by the higher L_m values, and thus the IIP NO LDA pressure stays close to the BL pressure, even if there is a greater force production compared to the BL case.

The differences in pressure development for the IIP LDA and IIP NO LDA cases can be explained as follows. During the filling phase, there is no activation, and pressure is mainly driven by the passive force, which is the same for both cases, as L_m is the same. During the isovolumic contraction, L_m does not vary and the force increase is similar for both cases. As ejection starts, L_m variations differ between IIP LDA and IIP NO LDA, and thus pressures in both cases are different, and in particular they reach different peak values.

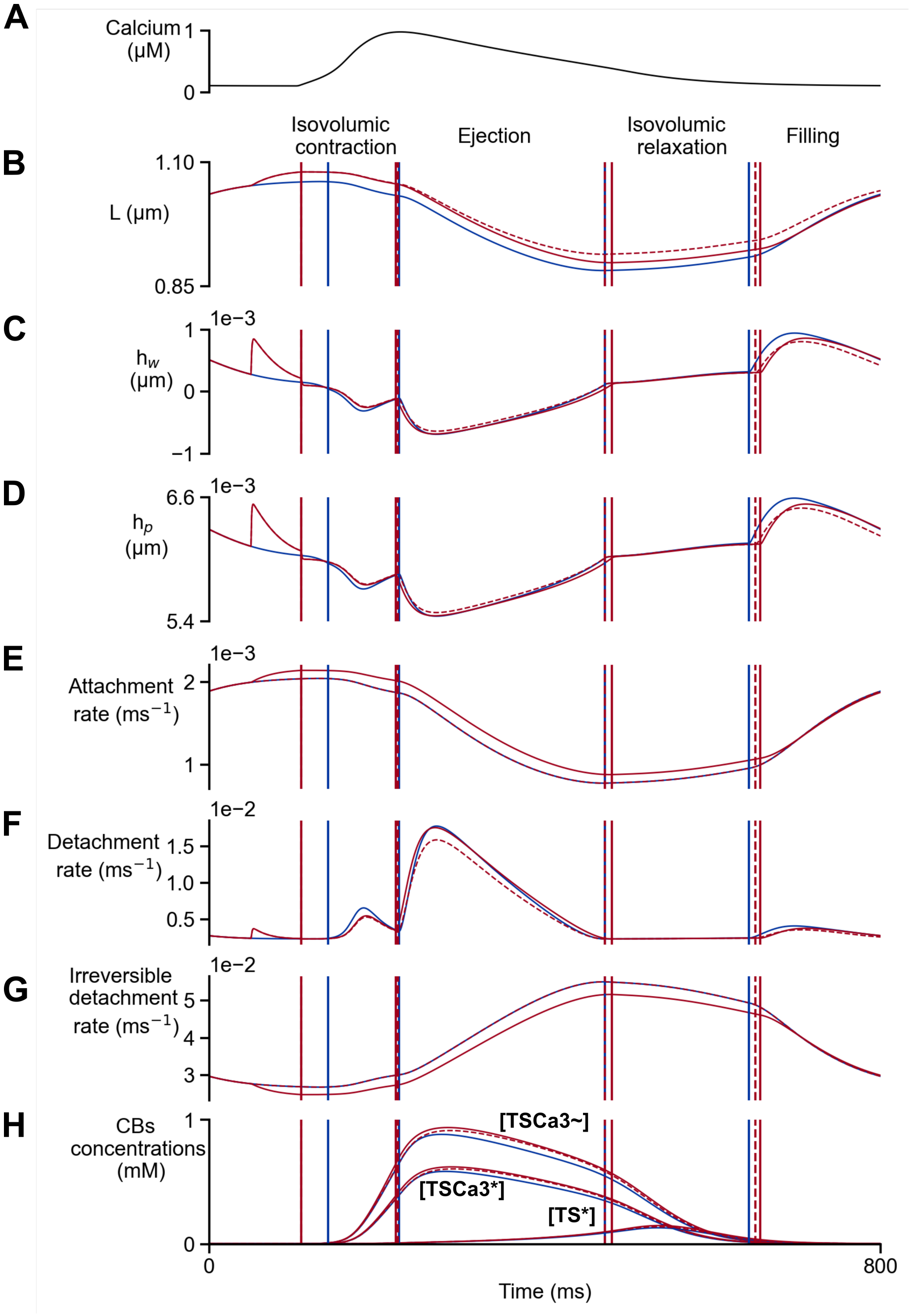

Fig A: Cellular variables during a heartbeat in the baseline case (solid blue), during an IIP protocol (solid red), and during an IIP protocol with NO LDA (dashed red). Vertical bars indicate intervals for the four phases of the cardiac cycle. A. Intracellular calcium concentration. B. Half-sarcomere length. C. Mean elongation of the crossbridges in the weak state. D. Mean elongation of the crossbridges in the power state. E. Attachment rate f. F. Detachment rate g. G. Irreversible detachment rate g_d. H. Attached crossbridges concentrations.

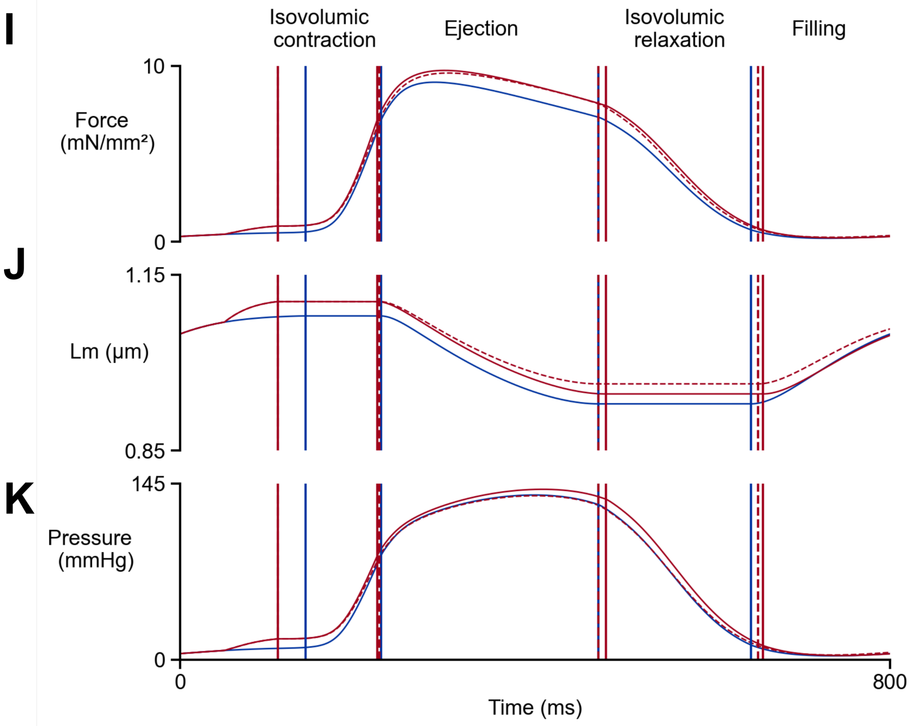

Fig A: (continued) I. Total force. J. Total half-sarcomere length. K. Ventricular pressure.

References

[1] J. A. Negroni and E. C. Lascano, “Simulation of steady state and transient cardiac muscle response experiments with a Huxley-based contraction model,” *J. Mol. Cell. Cardiol.*, vol. 45, no. 2, pp. 300–312, Aug. 2008, ISSN: 00222828.
